# Dimethyl malonate suppresses succinate dehydrogenase to alleviate ischemia/reperfusion-induced acute lung injury

**DOI:** 10.3389/fphar.2026.1746514

**Published:** 2026-09-17

**Authors:** Shih-En Tang, Shu-Yu Wu, Hsin-Ping Pao, Shi-Jye Chu, Wen-I Liao

**Affiliations:** 1 Division of Pulmonary and Critical Care, Department of Internal Medicine, Tri-Service General Hospital, National Defense Medical University, Taipei, Taiwan; 2 Institute of Aerospace and Undersea Medicine, National Defense Medical University, Taipei, Taiwan; 3 The Graduate Institute of Medical Sciences, National Defense Medical University, Taipei, Taiwan; 4 Division of Rheumatology, Immunology and Allergy, Department of Internal Medicine, Tri-Service General Hospital, National Defense Medical University, Taipei, Taiwan; 5 Department of Emergency Medicine, Tri-Service General Hospital, National Defense Medical University, Taipei, Taiwan

**Keywords:** acute lung injury, alveolar epithelial cell, dimethyl malonate, ischemia/reperfusion, succinate dehydrogenase

## Abstract

**Background:**

Ischemia/reperfusion (IR) injury is a pivotal contributor to acute lung injury (ALI) for which effective treatment options are currently lacking. Following ischemia, succinate accumulation and reoxidation by succinate dehydrogenase (SDH) during reperfusion induce excessive reactive oxygen species production, causing significant tissue damage. Dimethyl malonate (DMM), a competitive SDH inhibitor, has shown promise in treating various forms of IR-induced damage, but its effects on IR-induced ALI remain unexplored. To our knowledge, this is the first study to demonstrate in an *ex vivo* lung perfusion model that pharmacological inhibition of SDH via DMM alleviates IR-induced ALI.

**Methods:**

Isolated rat lungs subjected to ischemia (40 min) followed by reperfusion (60 min) were treated with various DMM concentrations (2 mM, 4 mM, and 8 mM) and 8 mM dimethyl succinate (DMS, a cell-permeable derivative of succinate). At the end of the experiment, bronchoalveolar lavage fluid and lung tissues were collected and analyzed. Target engagement was verified by measuring SDH enzymatic activity. Additionally, mouse lung epithelial (MLE)-12 cells underwent hypoxia-reoxygenation (HR) to investigate the cellular impact of DMM.

**Results:**

Metabolomic analysis revealed that succinate accumulated significantly in lung tissue after IR injury. DMM treatment effectively suppressed the IR-induced elevation of SDH enzymatic activity in both lung tissues and MLE-12 cells. Consequently, DMM dose-dependently reduced pulmonary edema and protein levels in bronchoalveolar lavage fluid. DMM also improved lung injury scores and attenuated proinflammatory cytokine production, apoptosis, and oxidative stress. These protective effects were associated with upregulated Nrf2/HO-1 activity and inhibited NF-κB signaling, all of which were reversed by DMS supplementation. Similar results were observed in MLE-12 cells following HR.

**Conclusion:**

These findings suggest that inhibiting SDH with DMM has considerable potential for mitigating IR-induced ALI. The therapeutic application of DMM could be a promising approach for alleviating IR-induced ALI severity.

## 1 Introduction

Acute lung injury (ALI) is a severe condition characterized by generalized inflammation in the lungs, increased permeability of the microvasculature, accumulation of fluid in the lungs, and damage to the alveoli. This condition can arise from ischemia and reperfusion (IR) following severe trauma, septic shock, pulmonary embolism or specific surgeries such as cardiopulmonary bypass and lung transplantation, ultimately leading to IR-induced ALI (Ferrari and Andrade, 2015). On a biochemical level, reperfusion stimulates the production of reactive oxygen species (ROS), thereby exacerbating oxidative stress within pulmonary tissue (Soares et al., 2019). Current management strategies for ALI primarily include lung-protective approaches and prone positioning. Despite the availability of these interventions, the mortality rate of this condition remains substantial (Bellani et al., 2016). Given this clinical challenge, there is an immediate need to explore innovative approaches for effectively and safely ameliorating ALI induced by IR.

Recent research findings suggest that succinate, an intermediate product of the citric acid cycle, accumulates during the ischemic phase, a period marked by oxygen deprivation (Zhang et al., 2018; Chouchani et al., 2014; Oh et al., 2023; Martin et al., 2019). Upon reperfusion, when oxygen is reintroduced, succinate undergoes reoxidation mediated by succinate dehydrogenase (SDH). This process subsequently leads to a significant increase in the production of ROS through reverse electron transport (RET) at mitochondrial Complex I (Zhang et al., 2018; Chouchani et al., 2014; Chouchani et al., 2016). A pioneering human study conducted by Kohlhauer et al. (2018) revealed a direct relationship between the amount of succinate released into the coronary artery and acute myocardial injury severity in patients with acute myocardial infarction. Interestingly, patients with pulmonary embolism exhibit elevated plasma succinate levels compared to healthy individuals (Cui et al., 2021). Wang and colleagues reported an increase in succinate levels in both lung tissue and serum following intestinal IR, which triggered alveolar macrophage polarization, induced alveolar epithelial apoptosis, and ultimately led to lung injury (Wang et al., 2023). Supporting the importance of this metabolic shift across different organs, a recent study demonstrated that reducing succinate accumulation during ischemia and preserving mitochondrial function upon reperfusion can alleviate kidney IR injury (Oh et al., 2023). In addition to its metabolic role, succinate acts as a signaling molecule modulating immune cell activation and cytokine production. Notably, the impact of succinate reoxidation may differentially affect the alveolar-capillary unit, contributing to microvascular permeability and epithelial barrier dysfunction.

Dimethyl malonate (DMM), a cell-permeable competitive inhibitor of SDH, has recently been employed to selectively inhibit SDH. DMM can suppress the production of TNF-α and IL-6, inhibit hepatocyte apoptosis, and notably increase survival rates in mice with lipopolysaccharide (LPS)/D-galactosamine-induced acute liver injury (Yang et al., 2019). In different physiological contexts, DMM has demonstrated therapeutic potential in rat models of collagen-induced arthritis. This compound mitigates joint swelling and inhibits both synovial tissue proliferation and inflammatory cell infiltration (Li et al., 2018). DMM also effectively suppresses pulmonary fibrosis, hypoxia-induced oxidative stress, and neuronal damage in neonatal mice (Wang et al., 2021; Zhang et al., 2022). Notably, in rats with brain and cardiac damage following resuscitation after cardiac arrest, DMM has been shown to primarily reduce ROS generation (Xu et al., 2018; Taghavi et al., 2022). This effect was also observed in rat models of brain IR injury, with DMM effectively reducing the extent of brain tissue infarction (Chouchani et al., 2014; Zhang Z. et al., 2021). Finally, in heart and kidney IR models, DMM was shown to minimize the damage associated with IR injury (Martin et al., 2019; Jespersen et al., 2020; Tonnesen et al., 2021; Kohlhauer et al., 2019; Carlström et al., 2024). This further highlights the potential therapeutic value of DMM in the clinical management of various IR-induced conditions.

IR injury-induced ALI remains a significant clinical challenge. Previous studies have highlighted the anti-inflammatory and antioxidant effect of DMM in IR injury to the brain, kidney and heart. However, the therapeutic potential of DMM against IR-induced ALI has yet to be investigated. Given the background presented above, we hypothesize that DMM may mitigate IR-induced ALI by inhibiting SDH and that DMM is a potential treatment for ALI. In this study, we aimed to determine whether the reversible inhibition of SDH via DMM can ameliorate IR-induced injury to isolated rat lungs and to investigate its effects on succinate metabolism and related oxidative and inflammatory signaling.

## 2 Methods

### 2.1 Animals

The animal protocols were approved by the Institutional Animal Care and Use Committee of the National Defense Medical Center under approval number IACUC-20–154. Male Sprague‒Dawley rats weighing approximately 350 ± 20 g were used in this study. The animals were treated humanely in according with the guidelines outlined by the National Institutes of Health (National Research Council, 1996). The rats were housed in a controlled environment at a standard temperature and humidity on a 12-h light‒dark cycle and provided unrestricted access to food and water.

### 2.2 Isolated rat lung perfusion model

To induce lung IR, we employed the approach outlined in our previous study, utilizing an *in situ* isolated rat lung perfusion model (Liao et al., 2021). In summary, Zoletil (50 mg/kg) was administered via intraperitoneal injection to anesthetize all the rats, and then tracheostomy was performed. Throughout the procedure, the rats were exposed to humidified air containing 5% CO_2_ and maintained at a positive end-expiratory pressure of 1 cm H_2_O. The mechanical ventilator settings were adjusted to a tidal volume of 3 mL and a breathing rate of 60 breaths per minute. Following median sternotomy, heparin (1 U/g of body weight) was injected into the right ventricle, and a cardiac puncture was performed to collect 10 mL of blood. A catheter made of polyethylene (PE)-10 material was inserted into the pulmonary artery, while simultaneously, another catheter made from PE-30 was advanced into the left atrium via the left ventricle. The side arm of the cannula allowed continuous measurement of both pulmonary venous pressure (PVP) and pulmonary arterial pressure (PAP). For perfusion of the isolated lung, a physiological salt solution (containing 119 mM NaCl, 4.7 mM KCl, 1.17 mM MgSO_4_, 22.6 mM NaHCO_3_, 1.18 mM KH_2_PO_4_, 1.6 mM CaCl_2_, 5.5 mM glucose, and 50 mM sucrose) supplemented with 4% bovine serum albumin was used. A 10 mL blood sample was aspirated from the animal and mixed with 10 mL of the physiological salt solution to prepare a 20 mL “half-blood” perfusate. DMM was administered by incorporation into this 20 mL perfusate, allowing direct delivery to the isolated lung during recirculation. A roller pump was used to maintain a consistent flow rate of 8 mL/min. Following the surgical procedure, the rats were placed on an electronic scale, allowing continuous monitoring of changes in lung weight.

### 2.3 Experimental design

In the study involving isolated rat lungs, the perfused lungs were allocated to seven experimental groups using random number-based randomization (n = 6 per group): 1) lungs subjected to sham procedures (control group), 2) lungs subjected to sham procedures and perfused with 8 mM DMM (Sigma Aldrich Company, St. Louis, MO, USA), 3) lungs subjected to IR (IR group), 4) lungs subjected to IR and perfused with 2 mM DMM, 5) lungs subjected to IR and perfused with 4 mM DMM, 6) lungs subjected to IR and perfused with 8 mM DMM, and 7) lungs subjected to IR were first perfused with 8 mM DMM for 10 min, followed by 8 mM dimethyl succinate (DMS, a cell-permeable derivative of succinate; Sigma Aldrich Company, St. Louis, MO, USA) for an additional 10 min before the onset of ischemia. MitoTEMPO was purchased from Sigma-Aldrich (St. Louis, MO, USA) and dissolved in sterile water. It was used at a final concentration of 10 μM in both the isolated rat lung model and MLE-12 cell experiments. The precise experimental timeline and tissue sampling points are illustrated in Supplementary Figure S1. The dosages of DMM and DMS used in this research were chosen on the basis of a previous study (Chouchani et al., 2014).

### 2.4 Lung weight (LW)/body weight (BW) and wet/dry (W/D) weight ratios

The LW/BW and W/D weight ratios were assessed as described previously (Liao et al., 2021). In brief, we promptly weighed the right middle lung after the surgical procedure to determine the wet LW. The LW/BW ratio was calculated by dividing the wet LW by the BW. Next, the right middle lung was placed in an oven and consistently heated at 60 °C for 48 h to obtain the dry LW. The W/D weight ratio was calculated by dividing the wet LW by the dry LW.

### 2.5 Vascular filtration coefficient (kf)

The K_f_ value was determined by examining the change in the LW due to the elevation of venous pressure, utilizing a well-established procedure described previously (Liao et al., 2021). The K_f_ value was calculated as the *y*-intercept (in g min^−1^) divided by the PVP (10 cm H_2_O) and LW and is expressed in whole units of gmin^−1^ cm H_2_O^−1^ × 100 g.

### 2.6 Assessment of CINC-1, IL-6 and TNF-α levels in bronchoalveolar lavage fluid (BALF)

We utilized commercially available mouse ELISA kits (R&D Systems Inc., Minneapolis, MN, USA) to assess the concentrations of CINC-1, IL-6 and TNF-α in the BALF. Quantification was carried out following the manufacturer’s protocols. The cytokine levels in the BALF were measured at the end of the 60-min reperfusion period.

### 2.7 Lung myeloperoxidase (MPO) activity (immunohistochemical analyses)

MPO serves a crucial function as an inflammatory enzyme that can trigger oxidative stress. MPO was detected using immunohistochemical staining according to previously described methods (Chu et al., 2021). In summary, formalin-fixed paraffin-embedded lung tissue sections were deparaffinized and subjected to antigen retrieval. To block endogenous peroxidase activity, the sections were incubated with a solution of 3% H_2_O_2_ and 100% methanol for 15 min. Immunostaining of lung sections was carried out using a rabbit polyclonal antibody against MPO (dilution: 1:100; Cell Signaling Technology). After two washes with phosphate-buffered saline (PBS), the slides were incubated with a secondary antibody (rat-specific horseradish peroxidase-conjugated) obtained from Nichirei Corporation (Tokyo, Japan) for 30 min. Horseradish peroxidase was detected via a chromogenic reaction involving diaminobenzidine for 3 min, and hematoxylin was used to counterstain the lung tissue sections.

### 2.8 Malondialdehyde (MDA), hydrogen peroxide (H_2_O_2_) and glutathione levels in lung tissue

Levels of MDA, H_2_O_2_, and glutathione in lung lysates were quantified using specific assay kits, including the Malondialdehyde Assay Kit (Cayman Chemical Inc., Ann Arbor, MI, USA), the Hydrogen Peroxide Assay Kit (Abcam Inc., Waltham, MA, USA), and the Glutathione Detection Assay Kit (Abcam Inc., Waltham, MA, USA). For MDA measurement, 25 mg of lung tissue was homogenized in 250 μL of T-PER™ Tissue Protein Extraction Reagent and centrifuged at 16,000 × g for 10 min at 4 °C. The supernatant was used for analysis as per the kit instructions. The mixture was boiled at 100 °C for 1 h and centrifuged at 1,600 × g for 10 min at 4 °C. Then, the mixture was kept at room temperature for 30 min. The absorbance of the supernatant was measured at 532 nm, and the MDA levels were expressed in nmol/mg protein. For H_2_O_2_ quantification, approximately 25 mg of lung tissue was homogenized in 150 μL assay buffer using a glass homogenizer. The homogenates were centrifuged at 13,000 × g for 5 min at 4 °C. The resulting supernatant was deproteinized and neutralized using perchloric acid and potassium hydroxide, respectively, according to the manufacturer’s instructions. The supernatant was used for the analysis. For the measurement of glutathione, 25 mg of lung tissue was homogenized in 150 μL of Cell Lysis Buffer on ice, followed by centrifugation at 14,000 × g for 10 min at 4 °C. The resulting supernatant was deproteinized and neutralized using perchloric acid and potassium hydroxide, respectively, according to the manufacturer’s instructions. The clarified samples were used for glutathione quantification. Because H_2_O_2_ is chemically reactive and can be influenced by tissue processing, homogenization, and enzymatic activity during sample preparation, the measured values should be interpreted as a bulk tissue estimate rather than a compartment-specific ROS readout; further limitations are discussed in the Discussion.

### 2.9 Terminal deoxynucleotidyl transferase dUTP nick end labeling (TUNEL) assay

TUNEL staining of 5 mm thick paraffin-embedded lung tissue sections was performed. The FragEL DNA Fragmentation Detection Kit and Fluorescent-TdT Enzyme supplied by Merck Millipore (Darmstadt, Germany) were used in strict accordance with the manufacturer’s guidelines. Specifically, for each experimental group, three lung tissue sections per animal were stained and analyzed. From each section, two fields were randomly selected at ×200 magnification using a fluorescence microscope (Leica DMi8, Germany). To eliminate observer bias, TUNEL-positive cell quantification was performed by an investigator blinded to group assignment.

### 2.10 Western blotting

Lung protein extracts (30 μg/lane) were separated by SDS-PAGE using 8%–10% gels for most targets, and 12% gels for cleaved caspase-3 to improve resolution of low-molecular-weight fragments. Following previously described methods (Liao et al., 2021), immunoblotting was conducted. The blots were probed with specific antibodies, such as anti-NF-κB p65, anti-phospho-NF-κB p65, anti-IκB-α, anti-phospho-IκB-α (dilution: 1:1,000, Cell Signaling Technology, Danvers, MA, USA), anti-Bcl-2 (dilution: 1:1,000, Bioss Inc., Woburn, Massachusetts, USA), anti-cleaved caspase-3 (dilution: 1:1,000, Cell Signaling Technology), anti-Nrf2 (dilution: 1:1,000, Bioworld Technology Inc., St. Louis Park, MN, USA), anti-HO-1 (dilution: 1:1,000, Enzo Life Science, Farmingdale, NY, USA), anti-lamin B1 (dilution: 1:200, Santa Cruz Biotechnology, Dallas, TX, USA), and anti-β-actin (dilution: 1:10,000, Sigma Chemical Company, St. Louis, MO, USA) antibodies. The ratio of the density of the target band to that of the reference protein band was calculated, and the target protein expression in the control group was set as 1.

### 2.11 Histopathological analysis

The neutrophil number in lung tissue and lung injury severity were assessed using established procedures (Liao et al., 2017). Briefly, lung tissues were fixed, cut into sections, and then subjected to hematoxylin and eosin (H&E) staining. Morphological examinations were carried out using light microscopy. Neutrophil infiltration in the airspace or vessel wall, as well as alveolar wall thickening, were evaluated in a minimum of 10 randomly chosen fields at ×200 magnification. Subsequently, lung injury severity was evaluated on a four-point scale: absent (0), slight (1), moderate (2), or intense (3). Two pathologists blinded to the experimental groups performed the scoring. To further minimize bias, tissue samples were coded prior to analysis, and the scores provided by both pathologists were aggregated to determine the lung injury score.

### 2.12 Hypoxia-reoxygenation (HR) modeled with mouse lung epithelial (MLE)-12 cells

MLE-12 cells were propagated in DMEM/F-12 medium supplemented with 10% fetal bovine serum and antibiotics such as penicillin (100 U/mL) and streptomycin (10 mg/mL). The cells were maintained at 37 °C in 5% CO_2_ in accordance with previously described methods (Chu et al., 2023). For HR modeling, MLE-12 cells were cultured under hypoxic conditions (1% O_2_, 5% CO_2_, 94% N_2_) for 2 h and then subjected to reoxygenation (5% CO_2_, 95% room air) for 1 h at 37 °C. Modeling was carried out in a humidified multigas incubator (APM-30D; ASTEL, Fukuoka, Japan). The cells were treated with control vehicle, 8 mM DMM, or 8 mM DMS. The dosage of DMM and DMS was determined through reference to a prior study (Chouchani et al., 2014). Next, proteins were extracted by lysing the cells, and the samples were subjected to Western blot analysis. Additionally, the supernatant was collected after HR to examine the level of keratinocyte chemoattractant (KC) using a mouse kC/CXCL-1 ELISA kit (R&D, Inc.).

### 2.13 Succinate assay

At the end of the 60-min reperfusion period, lung tissues were harvested, immediately snap-frozen in liquid nitrogen, and stored at −80 °C until analysis. For succinate extraction, 100 μL of 3 M perchloric acid was added to every 10 mg of frozen tissue powder, followed by homogenization using microtube pellet pestle rods. The homogenates were kept on ice for 10 min and centrifuged at 12,000 × g for 10 min at 4 °C. Subsequently, 10 μL of the resulting supernatant was diluted to 100 μL with water. An aliquot (10 μL) of the diluted sample was then mixed with water and succinic acid-d4 as an internal standard, vortexed for 10 s, and centrifuged at 12,000 × g for 5 min at 10 °C. The supernatant was transferred into HPLC vials for LC-MS/MS analysis. The succinate content was analyzed via a Waters ultrahigh-performance liquid chromatography (UPLC) system coupled with a SCIEX 4000 QTRAP LC-MS/MS system. The MS was operated in negative ion mode with multiple reaction monitoring. Chromatographic separation of succinate was achieved on a C18 column (100 × 2.1 mm, 2.6 μm particle size) using gradient elution with water as mobile phase A and methanol as mobile phase B. The flow rate was set at 0.4 mL/min. Succinic acid-d4 was used as the internal standard for quantitative analysis, and succinate concentrations were determined from the calibration curve and normalized to tissue weight.

### 2.14 Succinate dehydrogenase (SDH) activity assay

SDH activity was measured using a colorimetric assay kit (ab228560, Abcam, Cambridge, UK) according to the manufacturer’s instructions. Briefly, lung tissues or MLE-12 cells were homogenized in ice-cold SDH assay buffer and centrifuged at 10,000 × g to collect the supernatant. Samples were added to a 96-well plate, followed by the reaction mix containing assay buffer, electron probe, and substrate mix. Absorbance at 600 nm was measured immediately in kinetic mode at 25 °C for 10–30 min using a microplate reader. SDH activity was calculated from the change in absorbance over time based on the DCIP standard curve and normalized to protein concentration. Results are expressed as mU/mg protein.

### 2.15 Statistical analysis

Analysis of the data was carried out via GraphPad Prism nine statistical software (GraphPad Software, San Diego, CA, USA). The results are expressed as the means ± standard deviations. To compare the treatment and control groups, one-way analysis of variance (ANOVA) followed by Bonferroni’s *post hoc* test was conducted. Additionally, lung weight gain (LWG) across the groups was assessed via two-way repeated measures ANOVA followed Bonferroni’s *post hoc* test and p values less than 0.05 (p < 0.05) were considered to indicate statistical significance.

## 3 Results

### 3.1 DMM alleviates IR-induced acute pulmonary edema

Metabolomic analysis revealed that succinate levels in lung tissue surged by approximately 2,953% in the IR injury group compared to the control group (P < 0.05) (Figure 1A). Rats exposed to IR developed lung edema, as indicated by increases in LWG, the K_f_, the LW/BW ratio, the W/D weight ratio, and protein levels in BALF (Figures 1B–F). DMM significantly attenuated these increases in a dose-dependent manner. To confirm the harmful effects of succinate accumulation, DMS was co-administered with DMM (Oh et al., 2023). The results demonstrated that the protective effects of DMM on the lungs were diminished when DMS was introduced, reverting the physiological parameters toward the levels seen in the IR-only group.

**FIGURE 1:**
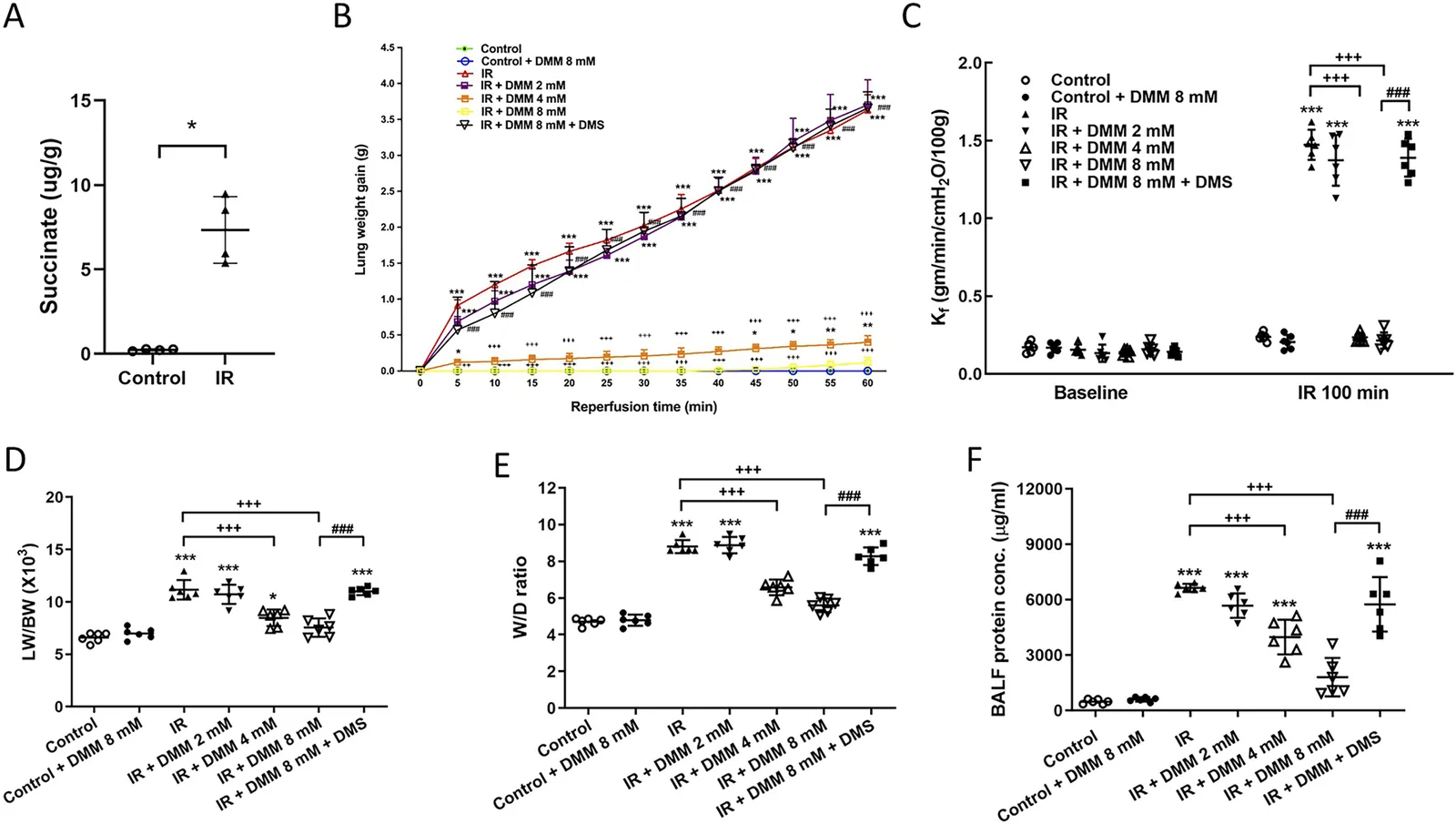
The beneficial effects of DMM on IR-induced acute lung edema. **(A)** Succinate levels in lung tissue were measured using UPLC-MS. The lung edema was assessed through **(B)** LWG **(C)** K_f_ **(D)** the LW/BW ratio **(E)** the W/D weight ratio, and **(F)** protein concentrations in BALF, all of which showed significant increases in the IR group. DMM significantly reduced these increases, with 8 mM DMM being the most effective dose for alleviating lung edema. However, this protective effect was reversed when co-perfused with 8 mM DMS. The measurement data are shown as the means ± SDs (n = 6 for each group). *p < 0.05, **p < 0.01, and ***p < 0.001 indicate statistical significance compared with the control group; + p < 0.05, ++ p < 0.01, and +++ p < 0.001 indicate statistical significance compared with the IR group; and #p < 0.05, ##p < 0.01, and ###p < 0.001 indicate statistical significance compared with the IR + DMM 8 mM group.

### 3.2 DMM decreases the IR-induced production of proinflammatory cytokines in the BALF and inhibits SDH activity in lung tissue

The concentrations of CINC-1, IL-6, and TNF-α in the BALF were significantly elevated in response to IR injury (Figures 2A–C). Treatment with 8 mM DMM effectively attenuated this IR-induced elevation in CINC-1, IL-6, and TNF-α levels, an effect that was abrogated by the co-administration of DMS. To further verify the pharmacological target of DMM, we measured the enzymatic activity of SDH in lung tissue homogenates. IR injury resulted in a significant increase in SDH activity by approximately 33.3% compared to the control group (Figure 2D). Following treatment with 8 mM DMM, this IR-induced elevation in SDH activity was markedly suppressed by approximately 22%. Notably, the inhibitory effect of DMM on SDH activity was reversed by co-perfusing with the competitive substrate DMS. These results provide compelling evidence that DMM exerts a suppressive effect on the inflammatory response by targeting SDH activity in the lungs following IR injury.

**FIGURE 2:**
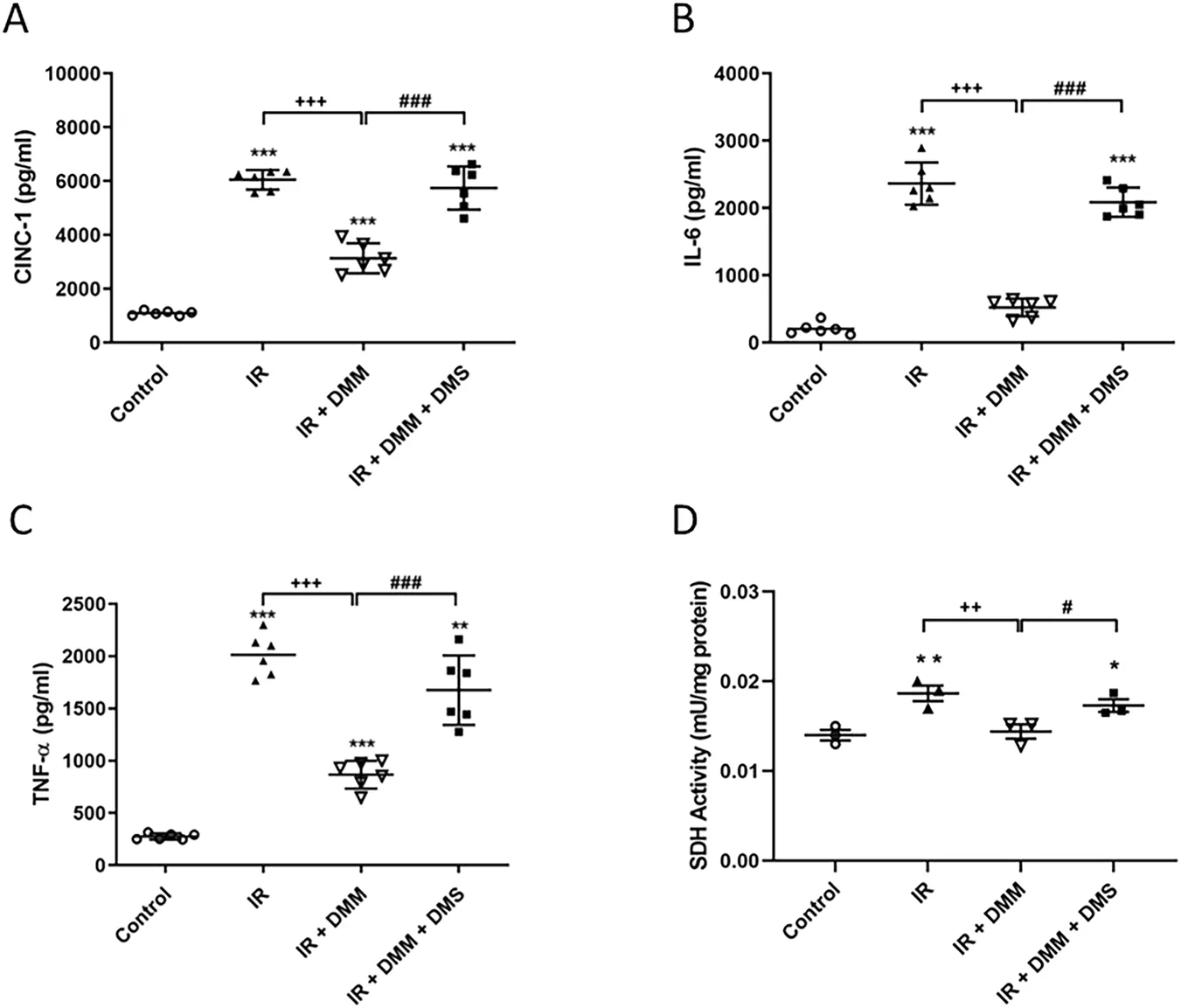
DMM reduced the IR-induced elevation of proinflammatory cytokine levels in BALF and SDH activity in lung tissue. **(A)** CINC-1 **(B)** IL-6, and **(C)** TNF-α levels in the BALF were measured using ELISA kits. In the IR group, levels of these cytokines were significantly higher compared to the control group. Treatment with 8 mM DMM markedly suppressed these increases following IR **(D)** SDH enzymatic activity was measured in lung tissue homogenates to evaluate target engagement. DMM significantly inhibited the IR-induced elevation of SDH activity. However, the anti-inflammatory effects and the inhibition of SDH activity of DMM were reversed when co-administered with 8 mM DMS. The measurement data are shown as the means ± SDs (n = 6 for each group). ***p < 0.001 indicate statistical significance compared with the control group, +++ p < 0.001 indicates statistical significance compared with the IR group, and ###p < 0.001 indicates statistical significance compared with the IR + DMM group.

### 3.3 DMM ameliorates neutrophil accumulation and histological alterations in lung tissue following IR

Compared to the control group, the IR group showed pronounced neutrophil infiltration in both intra-alveolar and interstitial regions, accompanied by significantly thickened alveolar walls (Figure 3A). Histological examination of lung tissue revealed substantially higher lung injury scores in IR-exposed rats compared to controls (Figure 3B). Administration of 8 mM DMM prior to IR exposure reduced inflammatory cell infiltration in the alveolar spaces, primarily by limiting neutrophil recruitment (Figure 3C). This reduction in lung injury scores, tissue damage, and neutrophil infiltration following DMM treatment was effectively reversed when co-administered with DMS.

**FIGURE 3:**
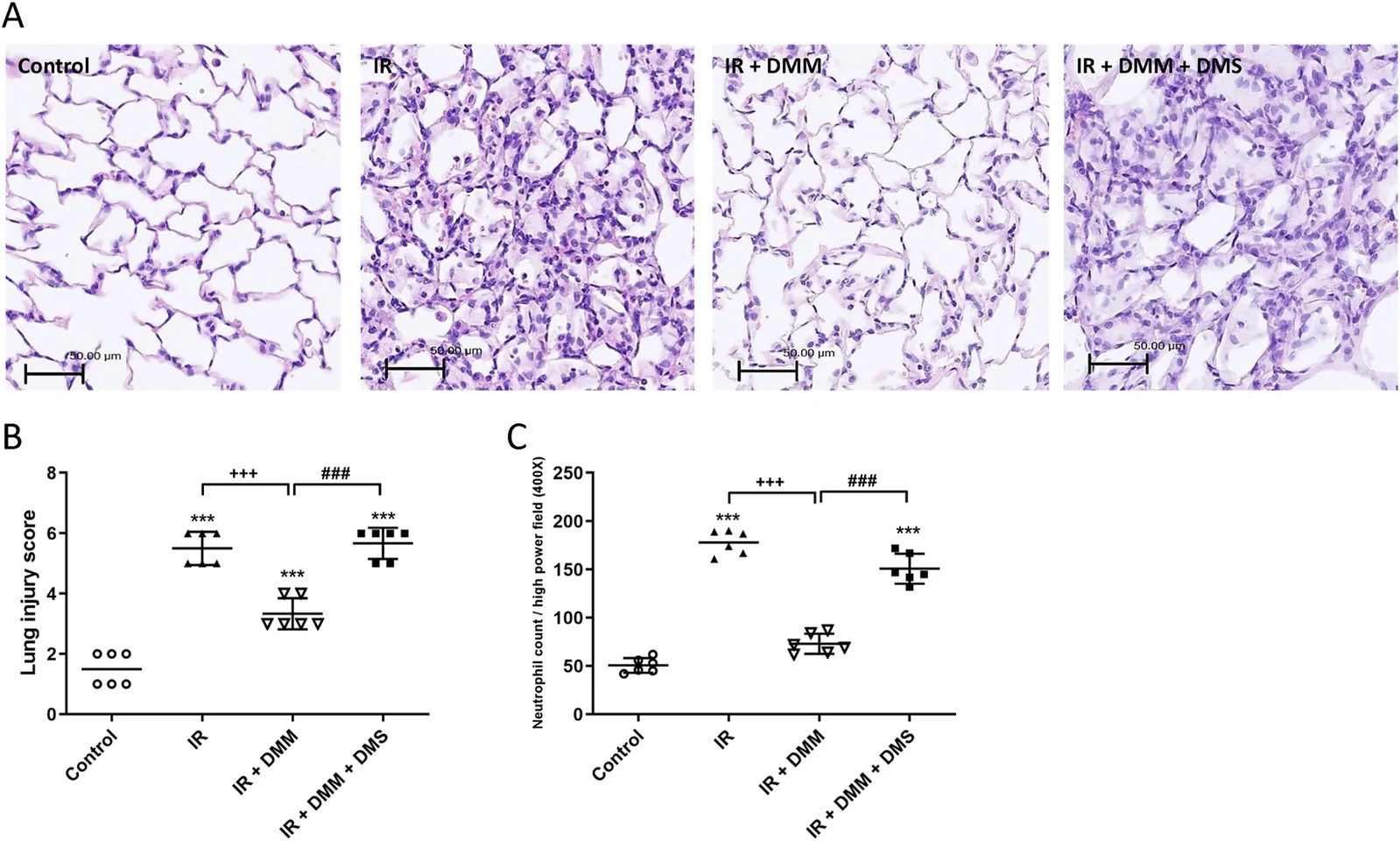
DMM alleviated lung pathology caused by IR. **(A)** H–E staining of lung tissue demonstrated increased alveolar wall thickness and enhanced neutrophil infiltration in the interstitial and alveolar spaces in the IR group compared to the control group. Treatment with 8 mM DMM mitigated the IR-induced alveolar damage **(B)** reduced lung injury scores, and **(C)** decreased the number of infiltrating neutrophils per high-powered field. However, the protective effects of DMM on the lung were effectively nullified by co-administration of 8 mM DMS. The measurement data are shown as the means ± SDs (n = 6 for each group). ***p < 0.001) indicates statistical significance compared with the control group, +++ p < 0.001 indicate statistical significance compared with the IR group, and ###p < 0.001 indicate statistical significance compared with the IR + DMM group.

### 3.4 DMM ameliorates oxidative stress in lung tissue caused by IR

IR pathogenesis is characterized by oxidative stress. To assess oxidative damage in lung tissue, the levels of MPO, MDA and H_2_O_2_, as markers of oxidative stress, were examined (Figures 4A–C). Conversely, the level of glutathione, which is critically involved in the elimination of many reactive species, was measured to assess antioxidant capacity (Figure 4D) (Forman et al., 2009). The results revealed considerable increases in the number of MPO-stained cells and MDA and H_2_O_2_ levels, as well as a decrease in glutathione levels, in lung tissue sections from the IR group compared with those from the control group. DMM significantly suppressed the rise in MPO, MDA, and H_2_O_2_ levels while preventing the reduction in glutathione, highlighting its effectiveness in mitigating oxidative damage. However, the beneficial effects of DMM were abolished when DMS was administered. Importantly, scavenging mitochondrial ROS with MitoTEMPO replicated the protective effects of DMM against IR-induced oxidative damage (Supplementary Figure S2).

**FIGURE 4:**
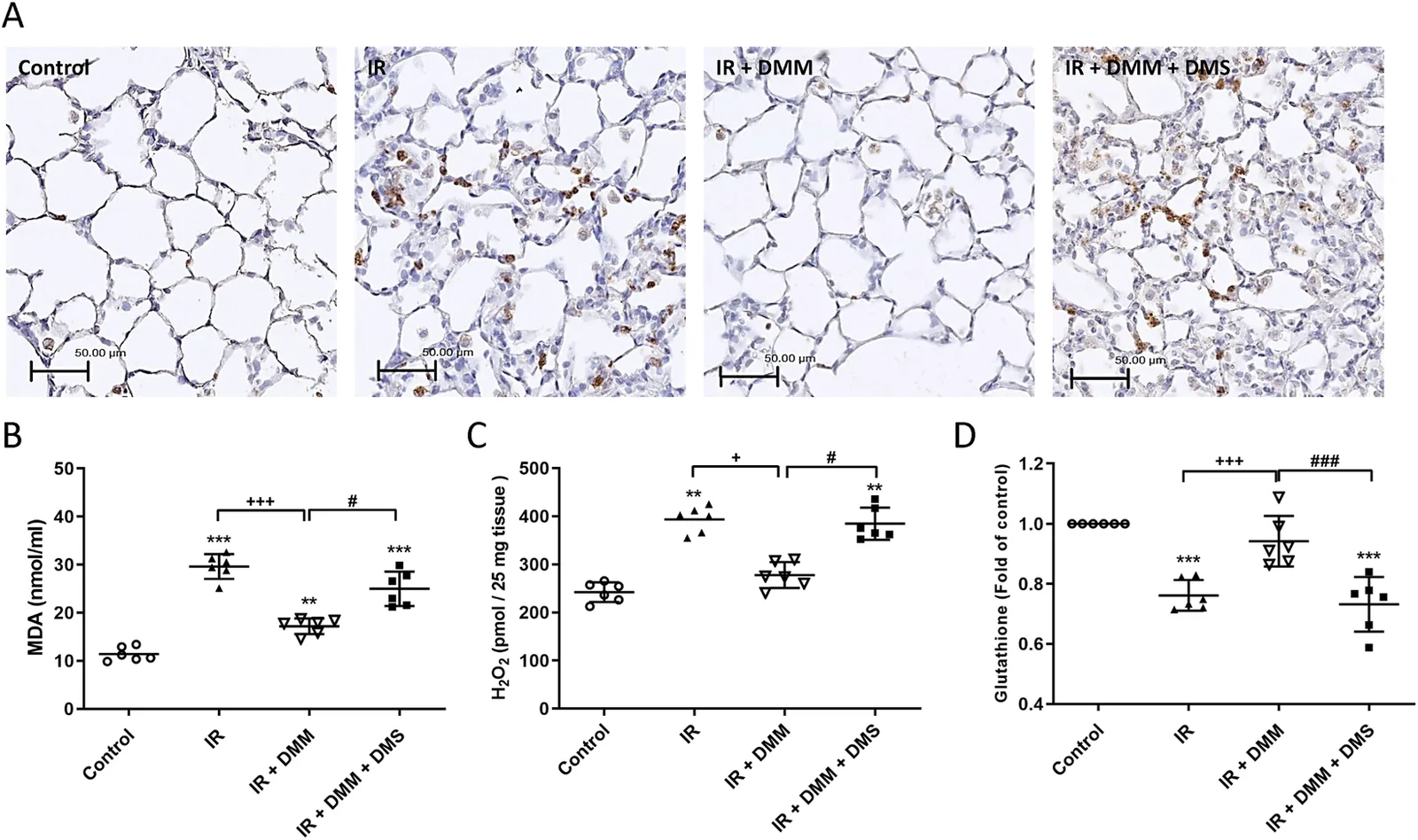
DMM attenuated IR-induced oxidative stress within lung tissue. IR resulted in a significant rise in **(A)** the count of MPO-positive cells, along with increased levels of **(B)** MDA and **(C)** H_2_O_2_, accompanied by a reduction in **(D)** glutathione levels. In contrast, 8 mM DMM significantly alleviated these effects of IR. However, the 8 mM DMS counteracted the beneficial effects of DMM. The measurement data are shown as the means ± SDs (n = 6 for each group). **p < 0.01 and ***p < 0.001 indicate statistical significance compared with the control group, + p < 0.05 and +++ p < 0.001 indicate statistical significance compared with the IR group, and #p < 0.05 and ###p < 0.001 indicate statistical significance compared with the IR + DMM group.

### 3.5 DMM attenuates IR-induced apoptosis in the lung

The function of Bcl-2, which is known for its antiapoptotic capabilities, as well as the activity of caspase-3 and the extent of TUNEL staining, which is an indicator of apoptosis during IR injury, were next assessed. Notably, the increases in the proportion of TUNEL-positive cells and the expression of cleaved caspase-3 and the reduction in Bcl-2 protein levels following IR injury were significantly mitigated by the administration of DMM (Figures 5A–D). However, these antiapoptotic effects of DMM were abrogated by the application of DMS.

**FIGURE 5:**
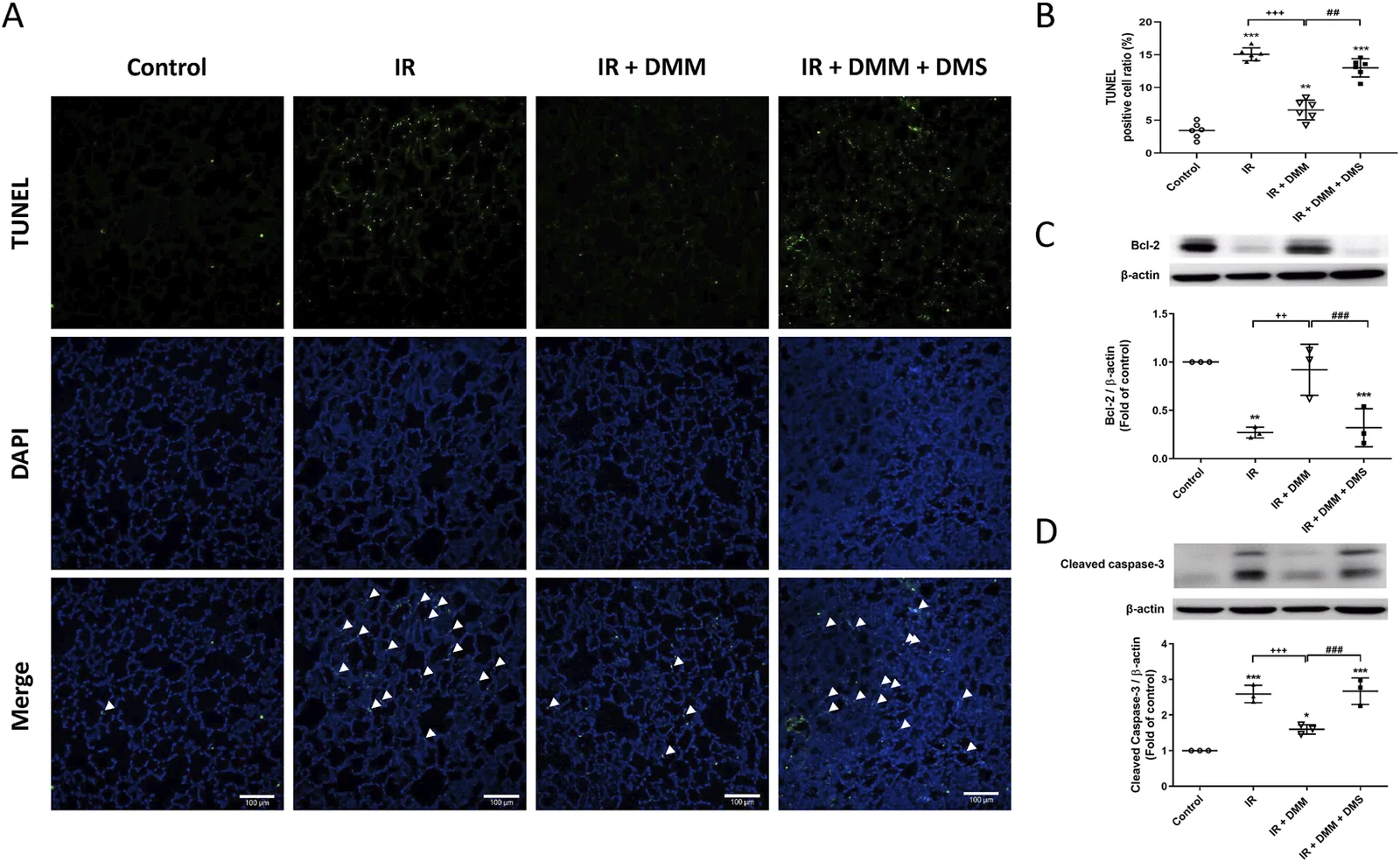
DMM ameliorated IR-induced apoptosis in lung tissue. **(A)** Lung tissue was subjected to a TUNEL staining. White arrowheads mark the location of TUNEL-positive cells **(B)** The bar diagram shows the quantitative changes in TUNEL staining. Furthermore, the protein levels of **(C)** Bcl-2 and **(D)** cleaved caspase-3 in the lung tissues were examined via Western blotting. Cytoplasmic protein levels were normalized to the level of β-actin as a loading control. Representative blots are shown. The measurement data are shown as the means ± SDs (n = 3 for each group). *p < 0.05, **p < 0.01, and ***p < 0.001 indicate statistical significance compared with the control group; ++ p < 0.01 and +++ p < 0.001 indicate statistical significance compared with the IR group; and ##p < 0.01 and ###p < 0.001 indicate statistical significance compared with the IR + DMM group.

### 3.6 DMM upregulates Nrf2/HO-1 and inactivates the NF-κB pathway in the lung

Previous studies have reported that IR decreases Nrf2/HO-1 expression and activates the NF-κB pathway (Dong et al., 2021). To investigate this further, we performed Western blot analysis to evaluate their expression in lung tissues. As illustrated in Figures 6A–D, our results demonstrated a significant increase in nuclear translocation of NF-κB and a concomitant reduction in nuclear Nrf2 expression following IR injury. Additionally, the expression of cytosolic HO-1 and IκB-α was markedly lower in the IR group compared to the control group. Importantly, DMM treatment effectively mitigated the IR-induced activation in NF-κB signaling and the reduction in Nrf2/HO-1 signaling. However, these beneficial effects of DMM were abolished by DMS.

**FIGURE 6:**
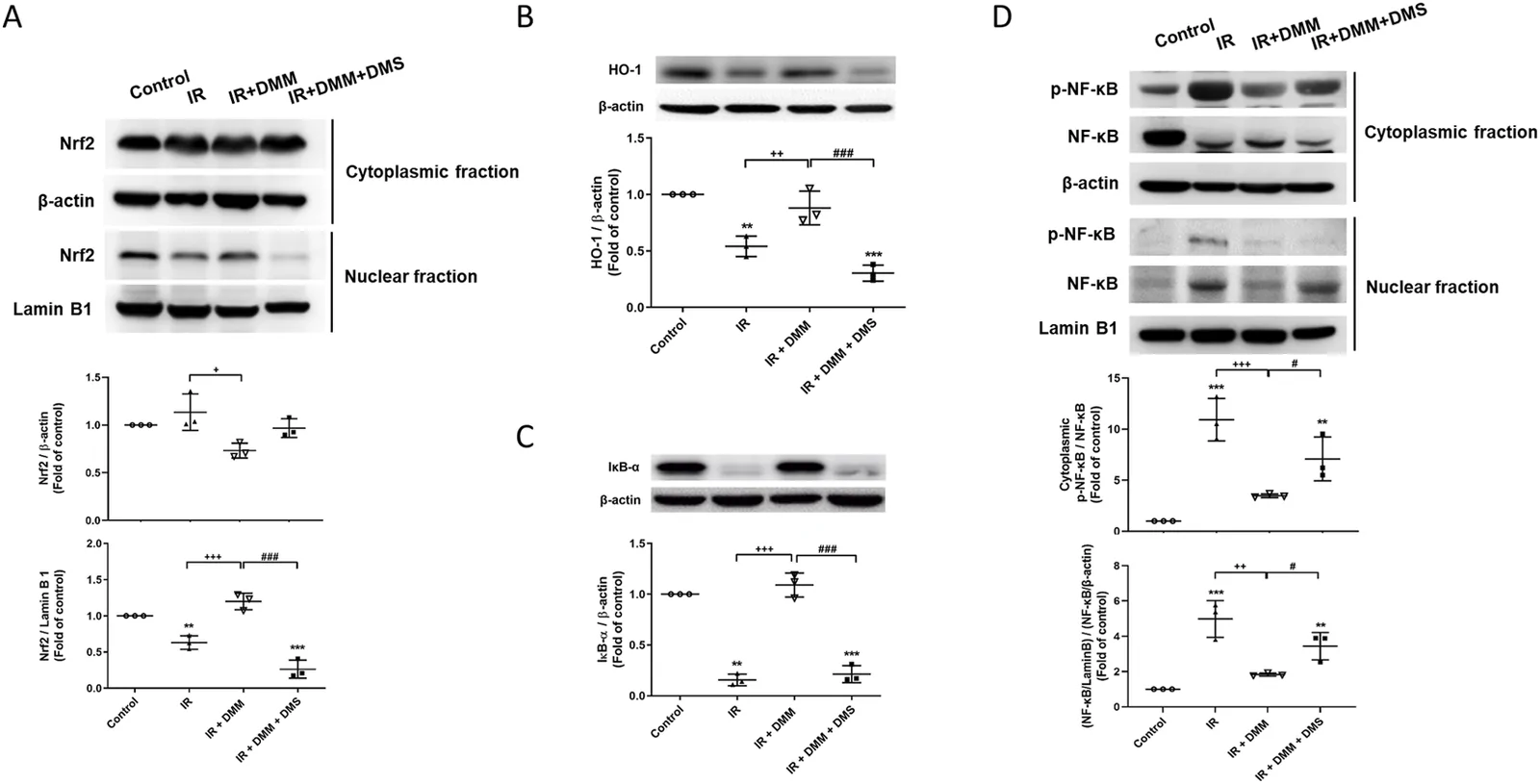
DMM activated the Nrf2/HO-1 and attenuated the NF-κB pathway in lung tissues. The figure shows the impact of DMM administration following IR-induced lung injury; specifically, DMM increased **(A)** nuclear Nrf2 **(B)** cytoplasmic HO-1, and **(C)** cytoplasmic IκB-α levels while concurrently decreasing the **(D)** cytoplasmic p-NF-κB levels and nuclear translocation of NF-κB. The protein markers lamin B1 and β-actin were used as normalization controls for nuclear and cytoplasmic protein levels, respectively. Representative Western blots are shown. The measurement data are shown as the means ± SDs (n = 3 for each group). *p < 0.05, **p < 0.01, and ***p < 0.001 indicate statistical significance compared with the control group; ++ p < 0.01 and +++ p < 0.001 indicate statistical significance compared with the IR group; and #p < 0.05, ##p < 0.01, and ###p < 0.001 indicate statistical significance compared with the IR + DMM group.

### 3.7 DMM inhibits SDH activity and decreases HR-induced NF-κB pathway activation and inflammatory cytokine production in MLE-12 cells

DMM effectively attenuated the increase in KC/CXCL-1 production in MLE-12 cells exposed to HR (Figure 7A). To verify the metabolic target *in vitro*, we measured SDH enzymatic activity in MLE-12 cell lysates. DMM treatment significantly reduced the HR-induced elevation of SDH activity, an effect that was outcompeted by the addition of the substrate DMS (Figure 7B). Furthermore, DMM also markedly increased the expression of IκB-α and reduced the levels of p-IκB-α and p-NF-κB in MLE-12 cells (Figures 7C,D). The beneficial effects of DMM on SDH activity, NF-κB signaling and the expression of KC/CXCL-1 in MLE-12 cells were counteracted by the administration of DMS. These results suggest that DMM may suppress the inflammatory response in alveolar epithelial cells by modulating SDH activity. Consistently, DMM treatment also suppressed the HR-induced elevation of SDH activity in RAW 264.7 macrophages (Supplementary Figure S3).

**FIGURE 7:**
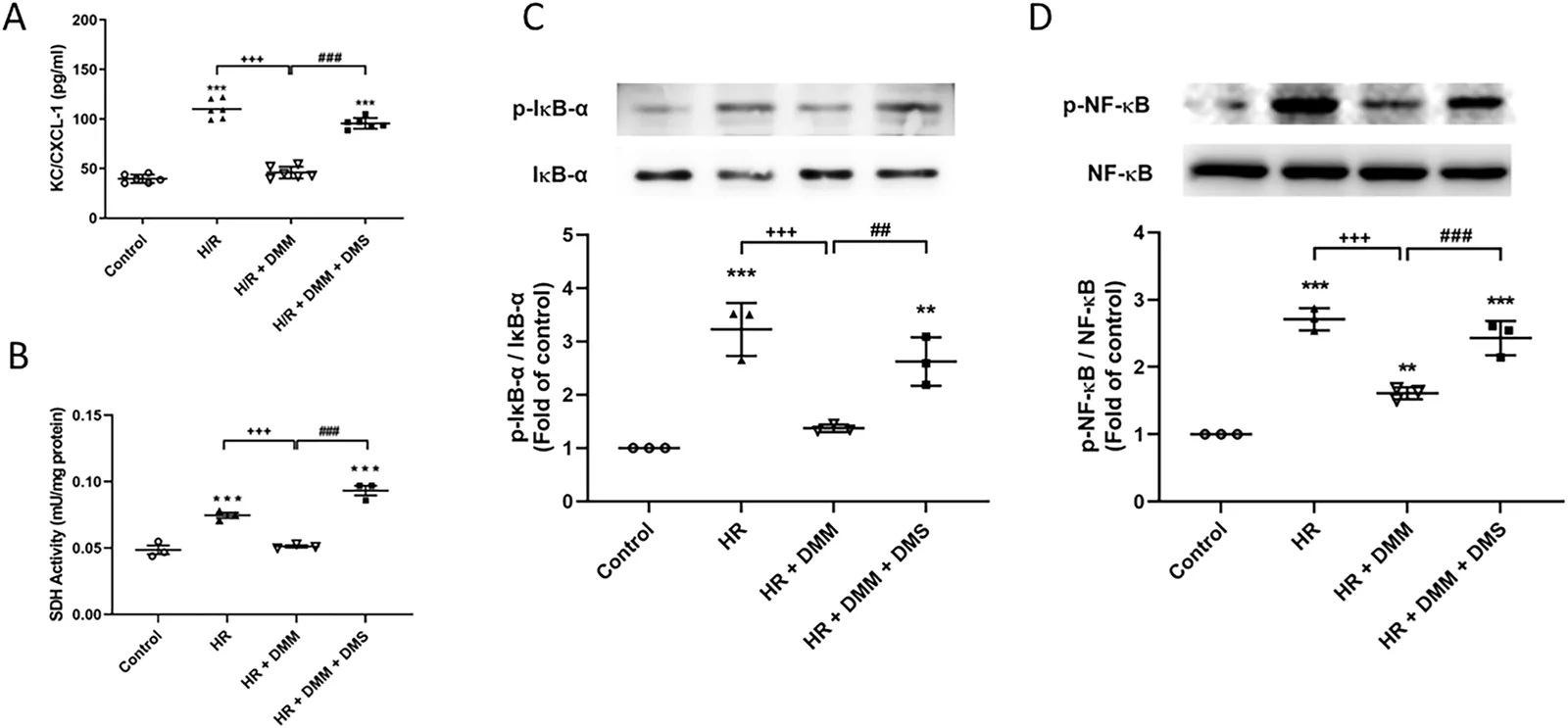
DMM attenuated HR-induced SDH activity, proinflammatory cytokine production and NF-κB signaling in MLE-12 cells. **(A)** The levels of KC/CXCL-1 in the cell culture supernatant were measured using ELISA **(B)** SDH enzymatic activity was measured in MLE-12 cell lysates to evaluate target engagement. DMM significantly inhibited the HR-induced elevation of SDH activity, which was reversed by DMS supplementation **(C,D)** Western blot analysis was used to evaluate p-IκB-α and IκB-α expression and p-NF-κB levels in MLE-12 cells subjected to 2 h of hypoxia followed by 1 h of reoxygenation. The measurement data are shown as the means ± SDs (n = 3 for each group). **p < 0.01 and ***p < 0.001 indicate statistical significance compared with the control group; + p < 0.05, and +++ p < 0.001 indicate statistical significance compared with the HR group; and ##p < 0.01 and ###p < 0.001 indicate statistical significance compared with the HR + DMM group.

## 4 Discussion

This study showed that DMM effectively alleviated lung inflammation, minimized pulmonary edema, lowered inflammatory cytokine levels, reduced oxidative stress and apoptosis, and improved histological lung injury scores in rats exposed to IR injury. In parallel, *in vitro* experiments revealed that DMM could reduce the levels of NF-κB p65 and inflammatory cytokine levels while simultaneously increasing the levels of IκB-α in MLE-12 cells subjected to HR. Moreover, the administration of DMS, a form of succinate capable of crossing cellular membranes, decreased the beneficial impact of DMM. Our findings suggest that DMM attenuates IR-induced ALI by reducing oxidative stress and inhibiting downstream inflammatory and apoptotic signaling.

The systematic metabolic partitioning of succinate during IR injury involves complex biochemical shifts. Under normoxic conditions, succinate is a key intermediate in the TCA cycle, where it is oxidized by SDH to fumarate. However, during the ischemic phase, succinate accumulates rapidly. While the mechanisms are debated, key proposed pathways include: (1) the reversal of the SDH reaction, where fumarate acts as an alternative electron acceptor and is reduced to succinate; and (2) aminotransferase anaplerosis, particularly from aspartate and glutamine, which provides significant carbon input into the canonical Krebs cycle under ischemic conditions (Zhang et al., 2018; Chouchani et al., 2014). Upon reperfusion, the sudden re-introduction of oxygen triggers a metabolic “bottleneck.” The accumulated succinate is rapidly oxidized by SDH, exceeding the capacity of the distal electron transport chain and forcing electrons to flow backward from Complex II to Complex I. This process, known as RET, is the primary driver of the massive mitochondrial ROS burst observed in our model (Zhang et al., 2018; Murphy and Hartley, 2018). In turn, ROS induce oxidative damage and initiate the activation of mitochondrial permeability transition pores, which leads to cellular death (Chouchani et al., 2016). To establish a direct causal relationship, we verified target engagement by measuring SDH enzymatic activity. DMM significantly suppressed the IR-induced surge in SDH activity in both lung tissues and MLE-12 cells, an effect competitively reversed by DMS. Furthermore, our supplementary experiments with MitoTEMPO demonstrated that scavenging mitochondrial ROS replicates the protective effects of DMM, reinforcing the role of the SDH-mtROS axis as the primary driver of injury.

By showing that DMM effectively reduces inflammatory markers in both our *ex vivo* lung model and *in vitro* MLE-12 cell model, our study highlights the broad-spectrum potential of SDH inhibition in stabilizing the entire alveolar-capillary unit during IR injury. The protective effects of SDH inhibition may manifest differently across various lung cell populations. In pulmonary vascular endothelial cells, succinate-driven ROS production has been shown to trigger the degradation of junctional proteins leading to increased microvascular permeability and the characteristic pulmonary edema seen in ALI (Atallah et al., 2024). Conversely, our findings, along with previous reports in alveolar epithelial cells, suggest that succinate reoxidation likely compromises the integrity of the alveolar-capillary barrier by inducing mitochondrial ROS-dependent epithelial apoptosis and the subsequent breakdown of the alveolar lining (Wang et al., 2025).

The production of ROS serves a critical function in immune cell activity. When macrophages are exposed to LPS, they transition to a glycolytic state and simultaneously increase succinate levels, resulting in increased mitochondrial oxidation of succinate through SDH. This process leads to the production of mitochondrial ROS (Mills et al., 2016). Furthermore, studies have linked elevated succinate levels to the exacerbation of inflammatory conditions, such as angiotensin II-induced aortic aneurysm and necrotizing enterocolitis, in which exogenous succinate increases the levels of IL-6, and TNF-α (Cui et al., 2021; Yan et al., 2022). In contrast, SDH inhibitors, such as DMM, have been shown to reduce myocardial infarction during cardiac IR injury (Valls-Lacalle et al., 2018). Our study demonstrated that IR led to succinate accumulation, a result aligning closely with the observations reported in previous research (Beach et al., 2020). Intriguingly, in direct opposition to succinate, DMM abrogated IR-induced oxidative stress and proinflammatory cytokine production. Moreover, this protective effect was abolished by DMS administration. This phenomenon may be attributed to the pivotal role of succinate reoxidation by SDH in mediating the IR-induced proinflammatory response in the lung. However, it should be noted that the use of DMS to restore succinate levels may also exert metabolic effects beyond mitochondrial oxidation. For instance, high intracellular succinate levels can potentially influence prolyl hydroxylase activity or signal through the succinate receptor SUCNR1. While the rapid onset of DMM’s protective effects and their reversal by DMS in our acute model point toward SDH as the primary mediator, these secondary metabolic actions of DMS cannot be completely discounted.

Mechanistically, the link between SDH-mediated succinate oxidation and inflammatory signaling likely involves ROS-dependent modulation of upstream regulatory proteins. Mitochondrial ROS can activate the IκB kinase (IKK) complex, leading to IκB degradation and NF-κB nuclear translocation; simultaneously, the cellular redox state influences Nrf2 stability via oxidative modifications of Keap1 (Bellezza et al., 2012; Dang et al., 2020). Our findings demonstrate that IR injury suppresses the cytoprotective Nrf2/HO-1 pathway while activating NF-κB-driven inflammation. By competitively limiting SDH-mediated succinate oxidation, DMM reduces the initial ROS pressure, thereby attenuating NF-κB activation while permitting or enhancing the Nrf2/HO-1 program. This dual regulatory role of DMM is strongly supported by our target engagement data: the protective effects were competitively reversed by DMS, and direct measurements confirmed significant SDH inhibition. Furthermore, the ability of MitoTEMPO to replicate DMM’s protection reinforces that these benefits are primarily driven by the suppression of SDH-mediated mitochondrial ROS rather than generalized metabolic disruption.

Notably, apoptosis is a key contributor to the pathogenesis of ALI triggered by IR (Chu et al., 2021). Oxidative stress activates cellular signaling pathways through transcription factors and proinflammatory cytokines, culminating in apoptosis. This process is crucial in the pathophysiology of pulmonary disorders (Liu et al., 2023). Recent research has shown that succinate stimulates the production of proinflammatory cytokines, specifically TNF-α. These cytokines consequently induce apoptosis in alveolar epithelial cells, contributing to the pathology of intestinal IR-induced ALI (Wang et al., 2023). As reported by Carlström et al. (2024), DMM treatment protects against apoptosis induced by IR in renal tissue. DMM effectively downregulates the expression of active caspase-3 and alleviates neuronal apoptosis associated with cardiac arrest and seizures (Xu et al., 2018; Zhang Y. et al., 2021; Zhang et al., 2020). In accordance with the aforementioned findings from the literature, our study revealed that DMM reduces the degree of apoptosis induced by IR in lung tissue.

Beyond succinate, SDH inhibition by DMM likely reduces downstream TCA cycle intermediates like fumarate and malate (Chouchani et al., 2014). While fumarate can theoretically exert cytoprotective effects via Nrf2 activation (de Oliveira, 2026), overwhelming evidence in IR models—including our own—suggests that preventing the SDH-mediated ROS burst is the dominant protective mechanism. The significant improvement in all primary indices of lung injury indicates that DMM’s anti-oxidative benefits far exceed any potential drawbacks from localized metabolite depletion. Regarding clinical translation, the stabilization of the alveolar-capillary unit through SDH inhibition represents a promising early-intervention strategy. In clinical scenarios such as lung transplantation, the lungs undergo distinct periods of ischemia followed by rapid reperfusion. Our results suggest that targeting the SDH-mediated ROS burst at the onset of reperfusion could mitigate the initial surge of oxidative stress and inflammatory signaling.

This study has several limitations. First, H_2_O_2_ levels were measured using an HRP-based assay on lung tissue homogenates. Although sensitive, this method may be influenced by the release of endogenous peroxidases during tissue processing (Murphy et al., 2022). Given the reactive nature of H_2_O_2_, these *ex vivo* measurements may not fully reflect *in vivo* conditions; thus, results should be interpreted with caution, and future studies using real-time probes (e.g., HyPer7) or LC-MS are warranted. Second, although the isolated perfused lung model provides a rigorously controlled platform to investigate organ-intrinsic mechanisms, it excludes systemic influences such as circulating immune cell recruitment and neurohumoral regulation. Consequently, our findings require further *in vivo* validation to fully account for the systemic inflammatory milieu characteristic of clinical ARDS. Third, while our data demonstrate a correlation between SDH inhibition and the modulation of NF-κB and Nrf2/HO-1 signaling, the precise causal linkages remain to be fully elucidated. We did not directly assess redox-sensitive phosphorylation events or use targeted genetic silencing to definitively trace the upstream signaling cascade. Thus, the observed dual regulation should be interpreted as correlative, necessitating future pathway-specific interventions to clarify the exact molecular transducers involved. Fourth, several pharmacological and metabolic uncertainties warrant consideration. We did not directly quantify the mitochondrial uptake of DMM or its pharmacokinetic profile under our specific perfusion conditions. Furthermore, while the competitive reversal of DMM’s effects by DMS strongly supports an SDH-dependent mechanism, we did not directly measure intracellular succinate levels following DMS administration. Our interpretation of this counter-action relies on the observed recovery of SDH enzymatic activity and the physiological reversal of the protective phenotype. Additionally, as a competitive inhibitor, DMM may influence the broader TCA cycle network beyond succinate or exert unanticipated off-target effects. Finally, as our metabolomic analysis was restricted to tissue harvested at the end of reperfusion, we did not perform time-resolved profiling during the ischemic phase. Future investigations utilizing subcellular fractionation, stable-isotope tracing (e.g., labeled DMS), and time-course targeted metabolomics are required to confirm precise organelle-level delivery and delineate the comprehensive metabolic trajectories in lung IR injury.

## 5 Conclusion

In conclusion, our study identifies the SDH-mediated mitochondrial ROS burst as a primary driver of lung IR injury and demonstrates the therapeutic potential of DMM in preserving alveolar-capillary integrity. By mitigating the initial metabolic “bottleneck,” SDH inhibition offers a promising early-intervention strategy for clinical scenarios such as lung transplantation. These findings provide a strong mechanistic foundation for developing mitochondria-targeted therapies to reduce the severity of ALI.

## Data Availability

The data presented in the study are deposited in the Metabolomics Workbench repository (https://www.metabolomicsworkbench.org/), accession number ST005148 (DOI: http://dx.doi.org/10.21228/M8JC5J).
