# Supplementary material for: Dimethyl malonate suppresses succinate dehydrogenase to alleviate ischemia/reperfusion-induced acute lung injury
Source: Front Pharmacol. 2026 Sep 17;17:1746514. doi: 10.3389/fphar.2026.1746514 (PMC13626917; doi:10.3389/fphar.2026.1746514)

Lung tissue

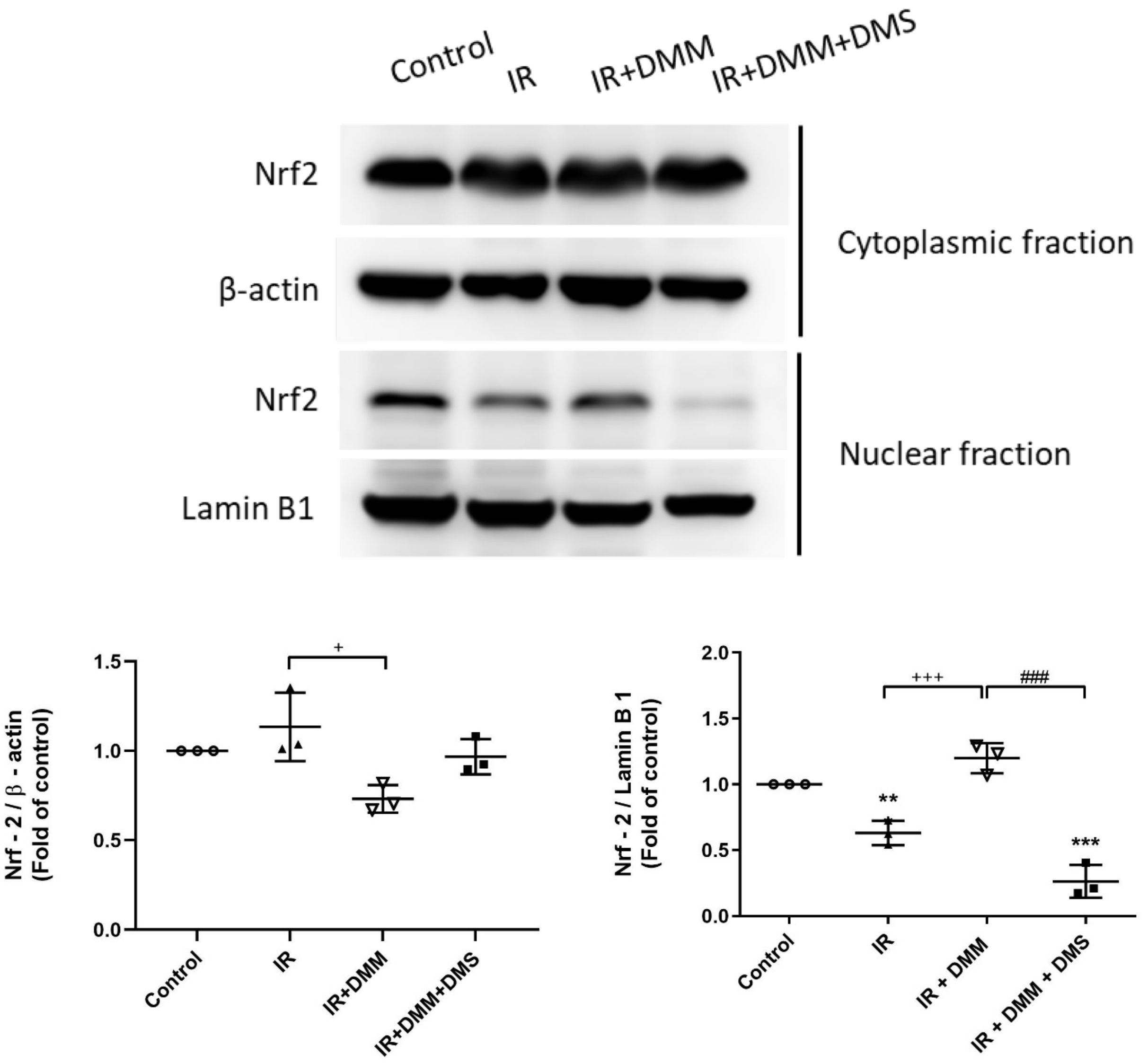

Cytoplasmic fraction

Nuclear fraction

Lung tissue

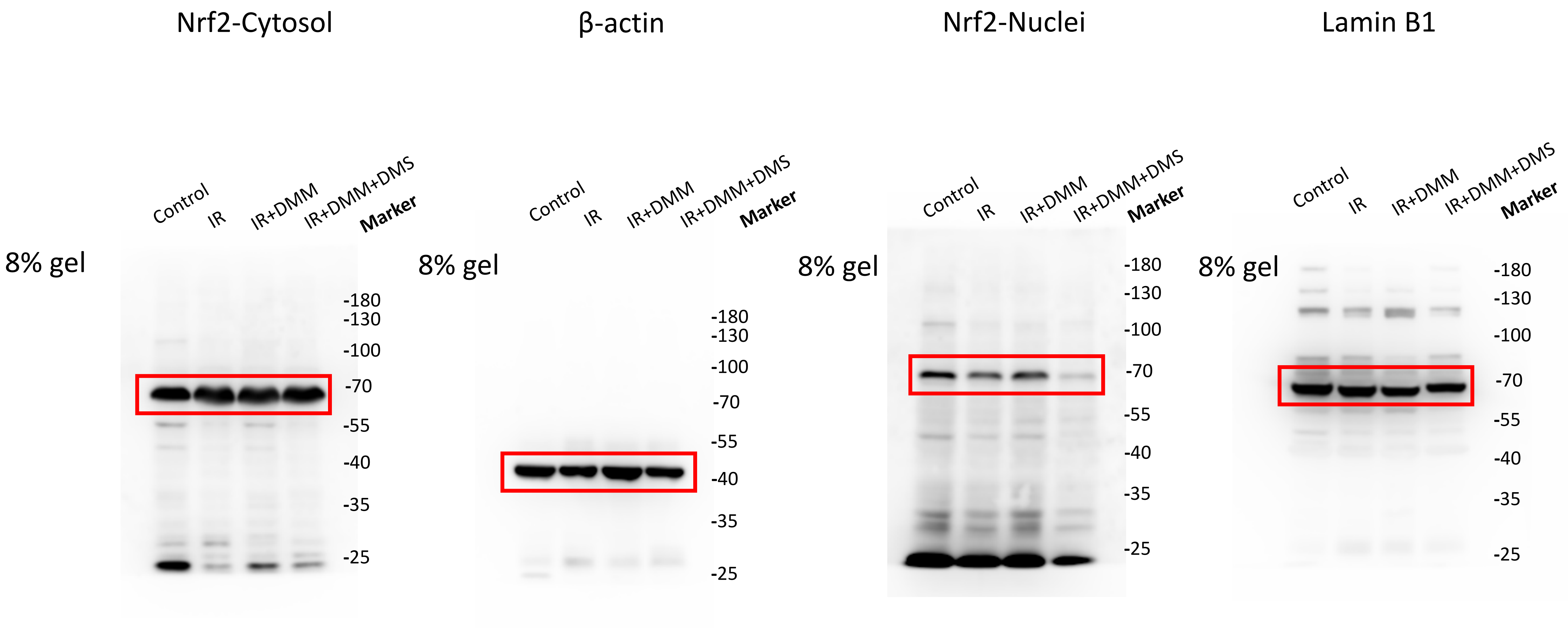
Nrf2-Cytosol
β-actin
Nrf2-Nuclei
Lamin B1
Control
IR
IR+DMM
IR+DMM+DMS
Marker
8% gel
-180
-130
-100
-70
-55
-40
-35
-25

Lung tissue

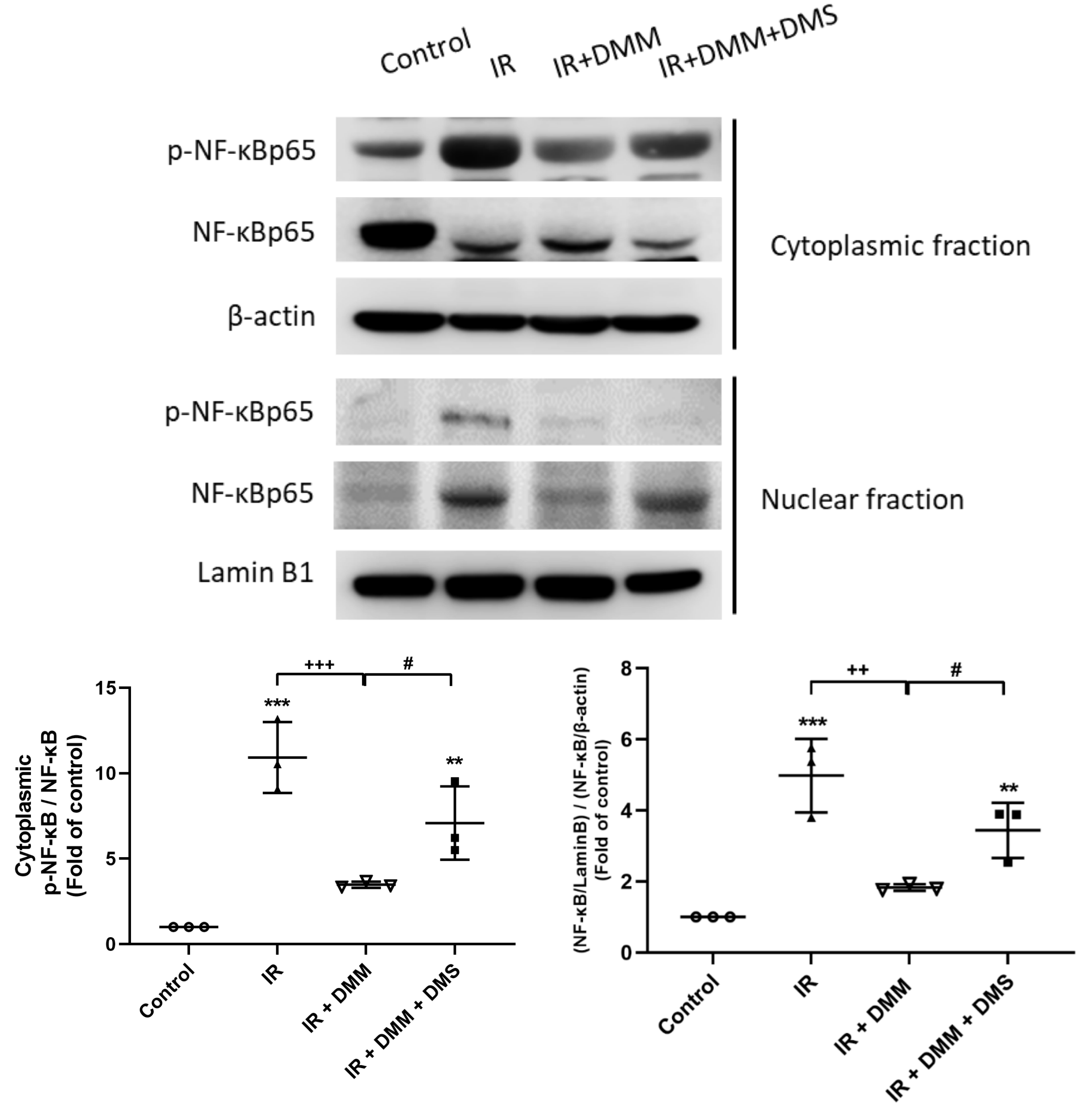

Cytoplasmic p-p65 / p65 ratio

(Tp65/Lamin B) / (Tp65/actin) ratio

Lung tissue

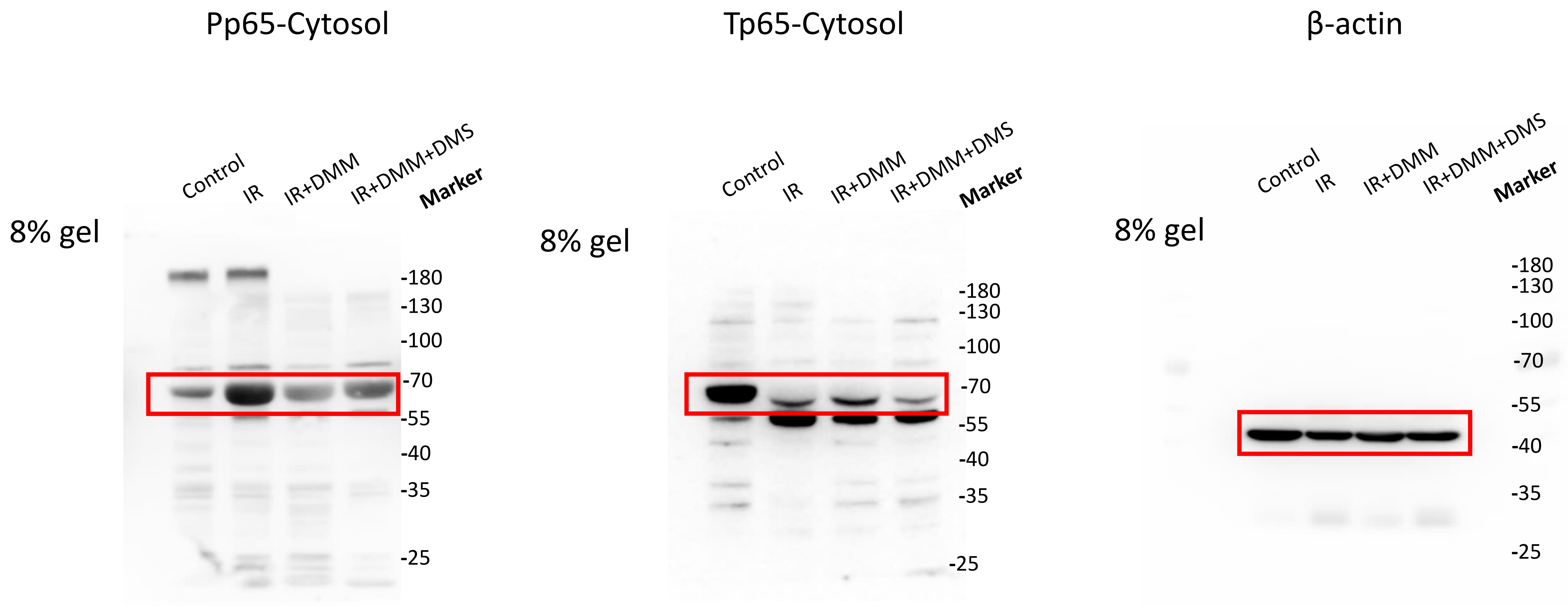
Pp65-Cytosol
Tp65-Cytosol
β-actin
8% gel
Control
IR
IR+DMM
IR+DMM+DMS
Marker
-180
-130
-100
-70
-55
-40
-35
-25

Lung tissue

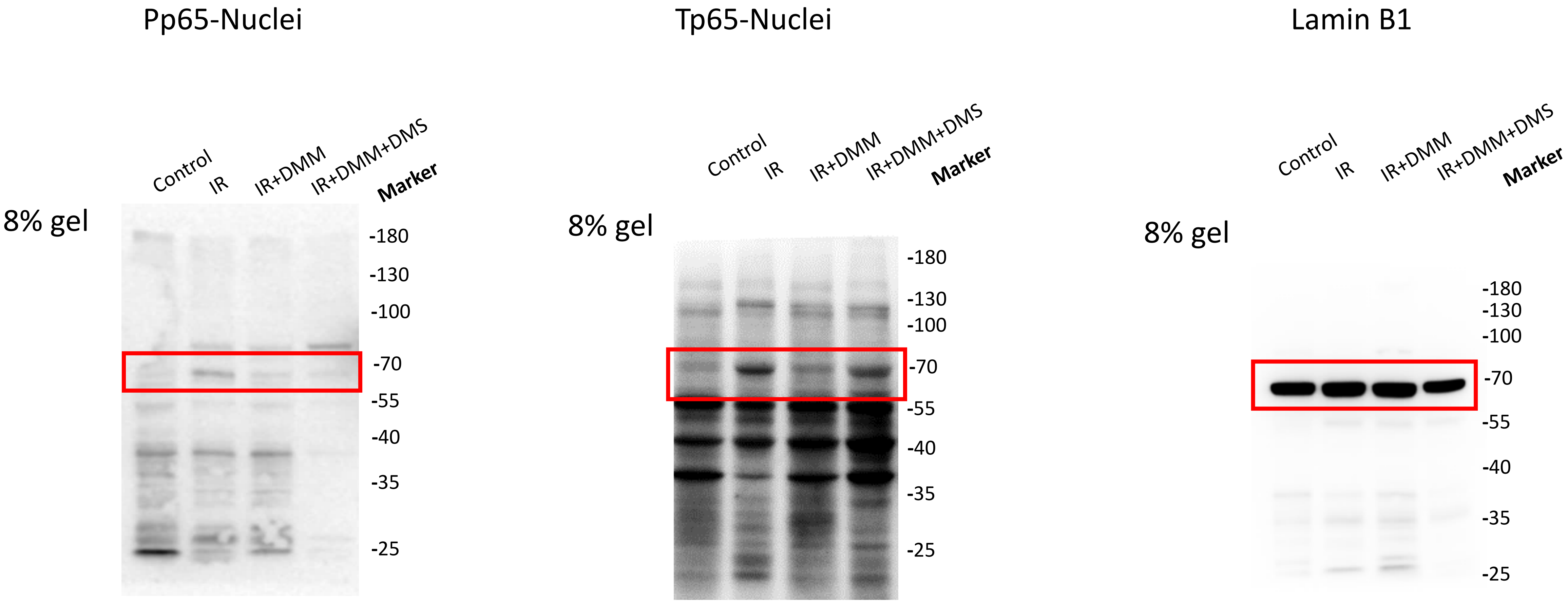

Pp65-Nuclei
Control
IR
IR+DMM
IR+DMM+DMS
Marker
8% gel
-180
-130
-100
-70
-55
-40
-35
-25
Tp65-Nuclei
Control
IR
IR+DMM
IR+DMM+DMS
Marker
8% gel
-180
-130
-100
-70
-55
-40
-35
-25
Lamin B1
Control
IR
IR+DMM
IR+DMM+DMS
Marker
8% gel
-180
-130
-100
-70
-55
-40
-35
-25

Lung tissue

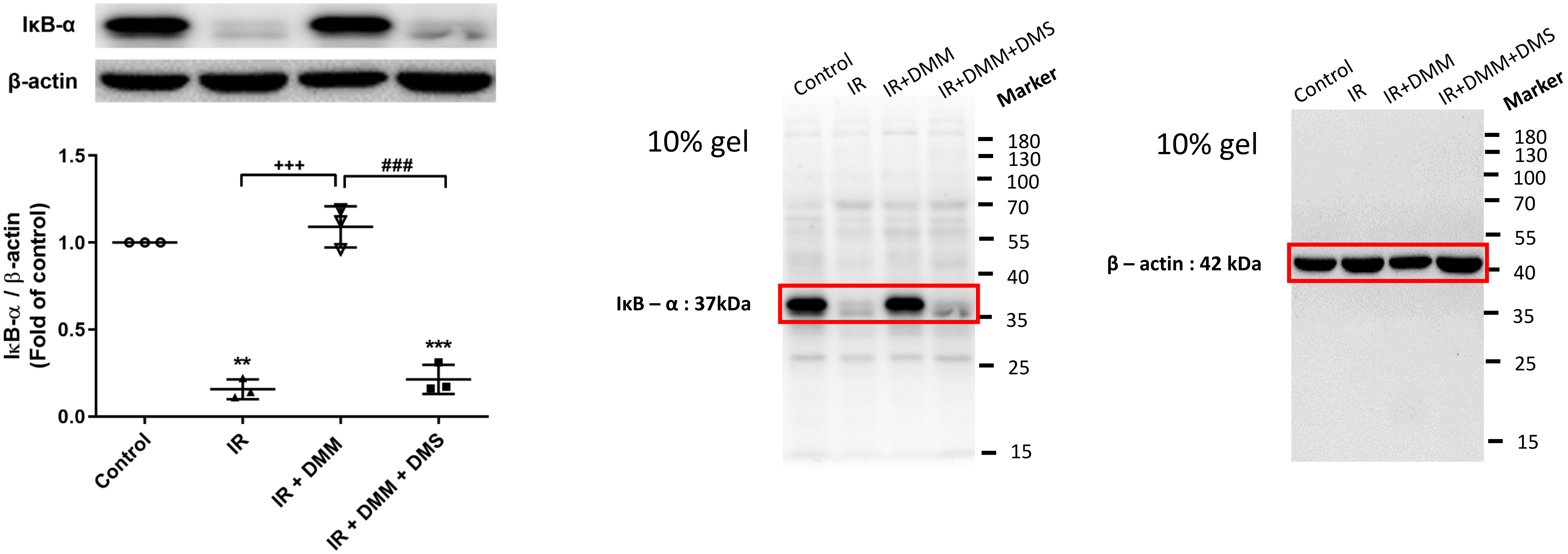

IκB-α
β-actin
IκB-α / β-actin (Fold of control)
1.5
1.0
0.5
0.0
+++
###
**
***
Control
IR
IR + DMM
IR + DMM + DMS
10% gel
Control
IR
IR+DMM
IR+DMM+DMS
Marker
180
130
100
70
55
40
35
25
15
IκB – α : 37kDa
10% gel
Control
IR
IR+DMM
IR+DMM+DMS
Marker
180
130
100
70
55
40
35
25
15
β – actin : 42 kDa

Lung tissue

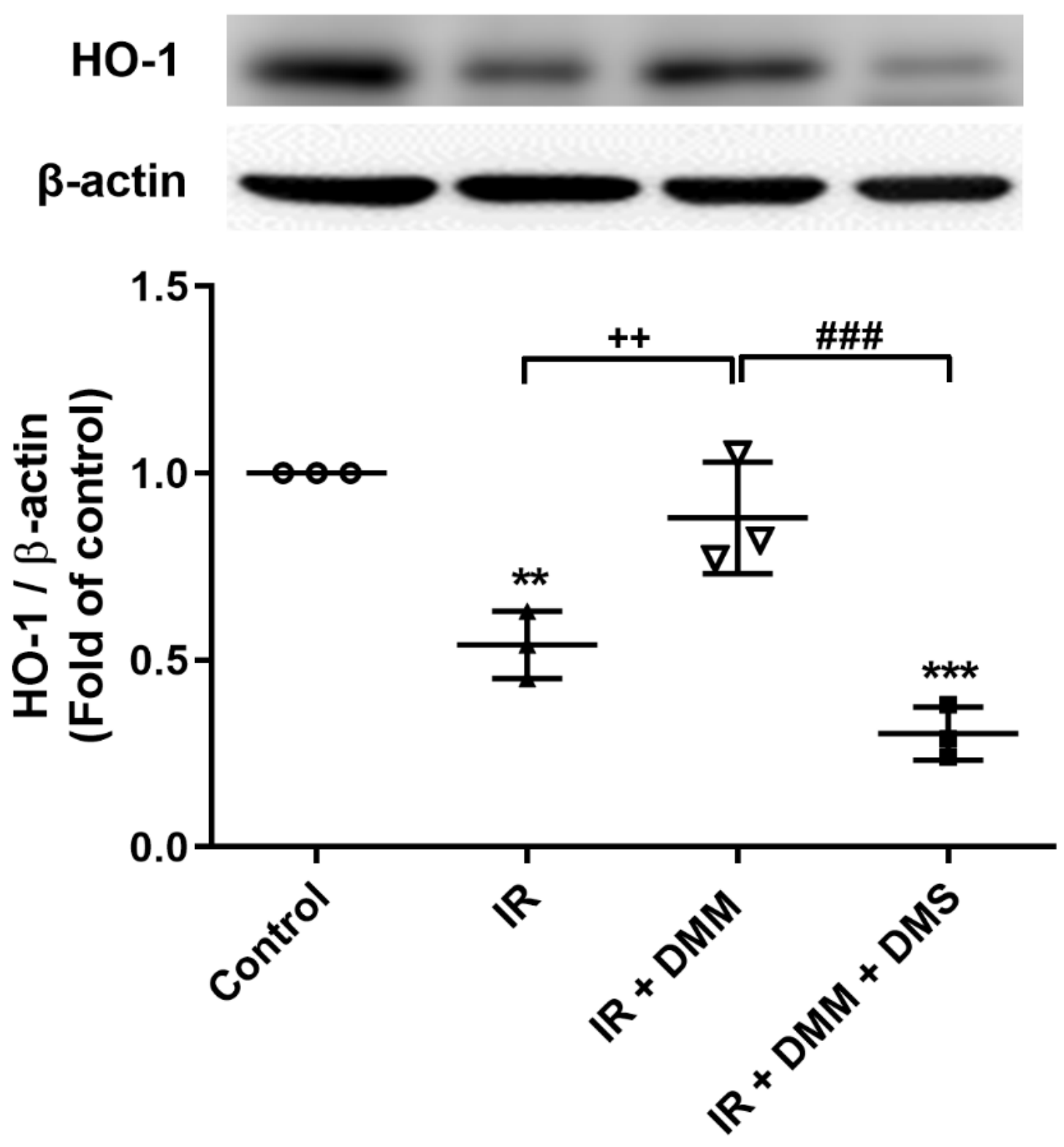

HO-1
β-actin
HO-1 / β-actin (Fold of control)
1.5
1.0
0.5
0.0
++
###
**
***
Control
IR
IR + DMM
IR + DMM + DMS

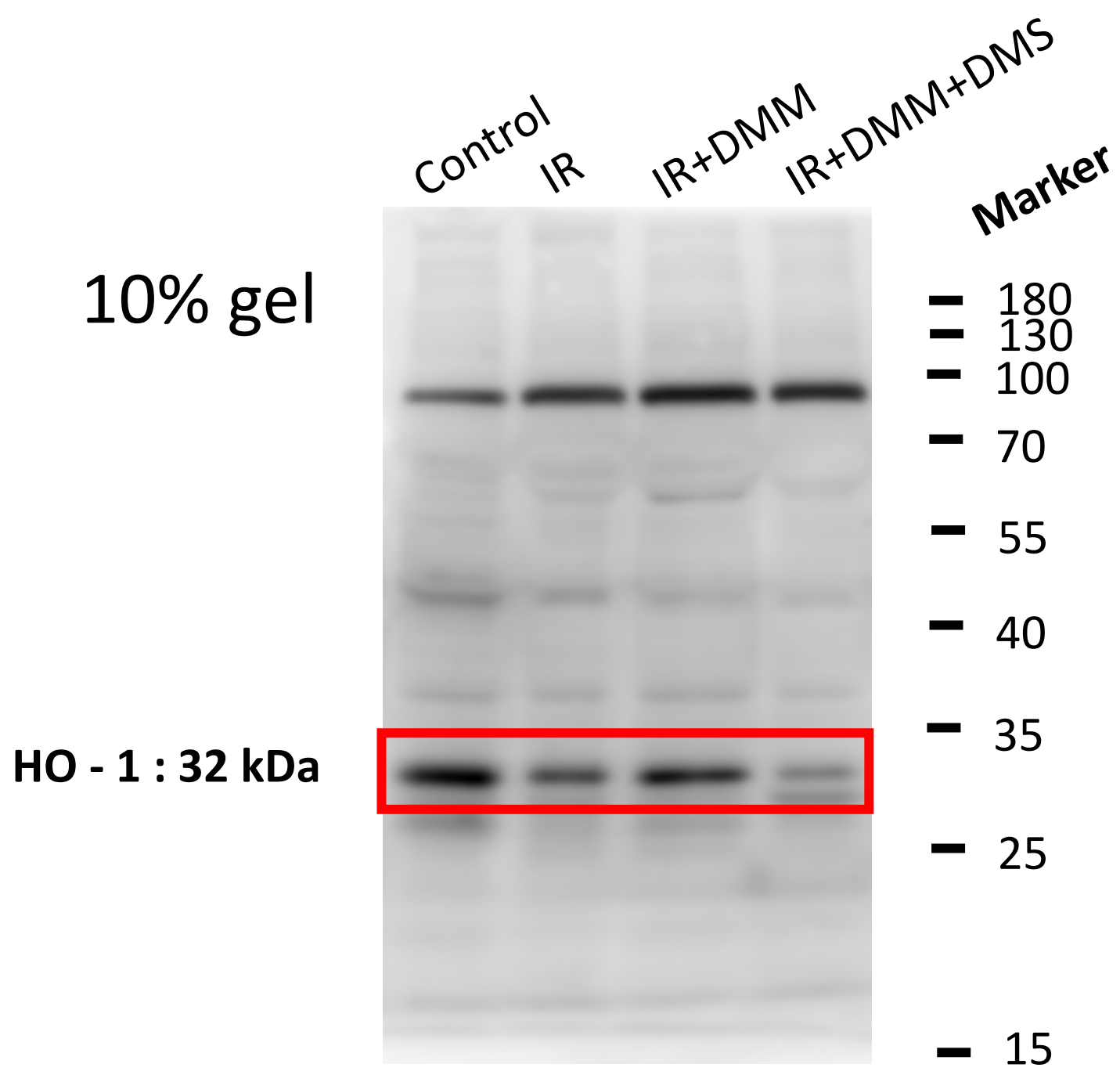

10% gel
Control
IR
IR+DMM
IR+DMM+DMS
Marker
180
130
100
70
55
40
35
25
15
HO - 1 : 32 kDa

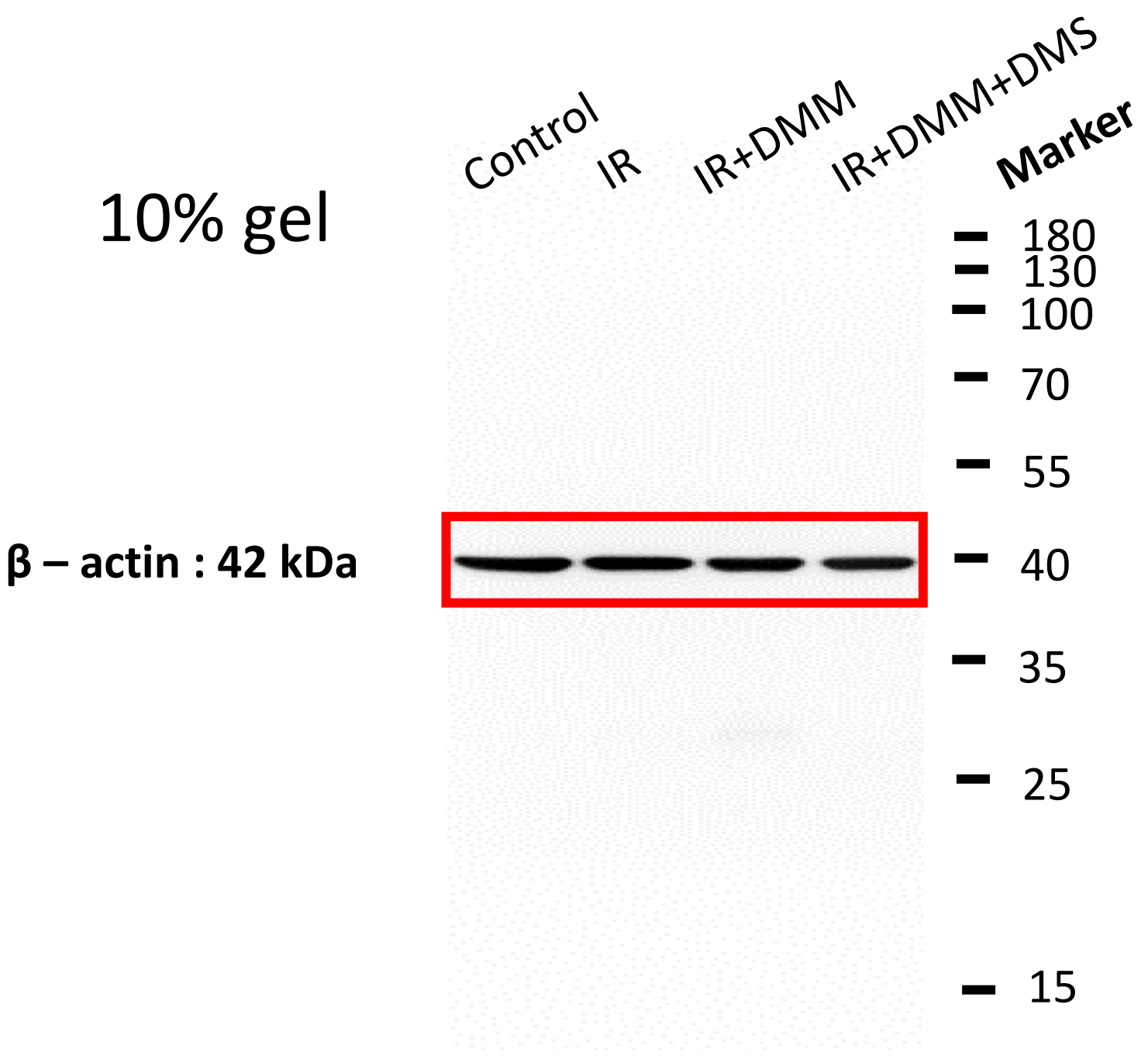

10% gel
Control
IR
IR+DMM
IR+DMM+DMS
Marker
180
130
100
70
55
40
35
25
15
β – actin : 42 kDa

Lung tissue

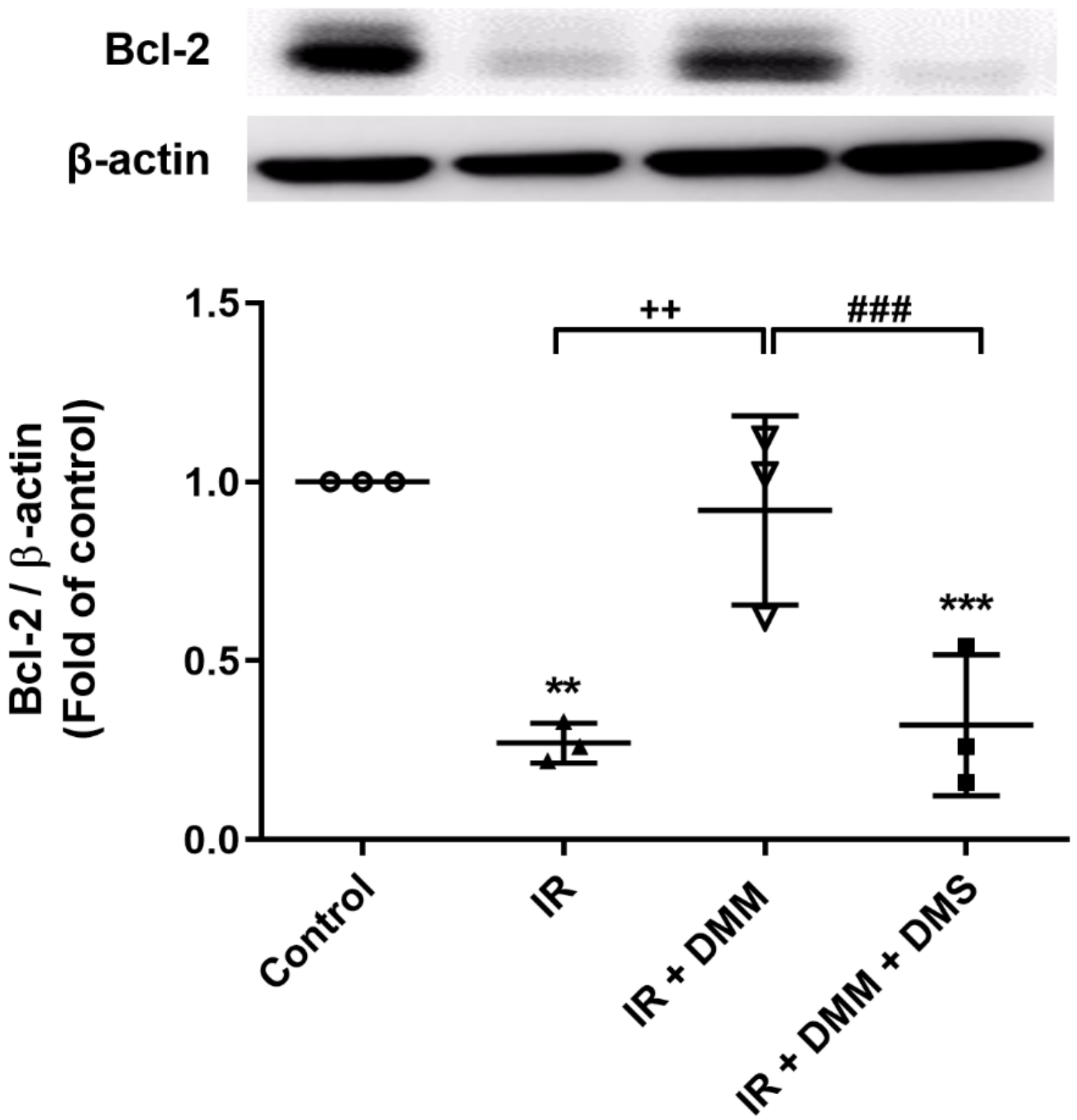

Bcl-2
β-actin
Bcl-2 / β-actin
(Fold of control)
1.5
1.0
0.5
0.0
++
###
**
***
Control
IR
IR + DMM
IR + DMM + DMS

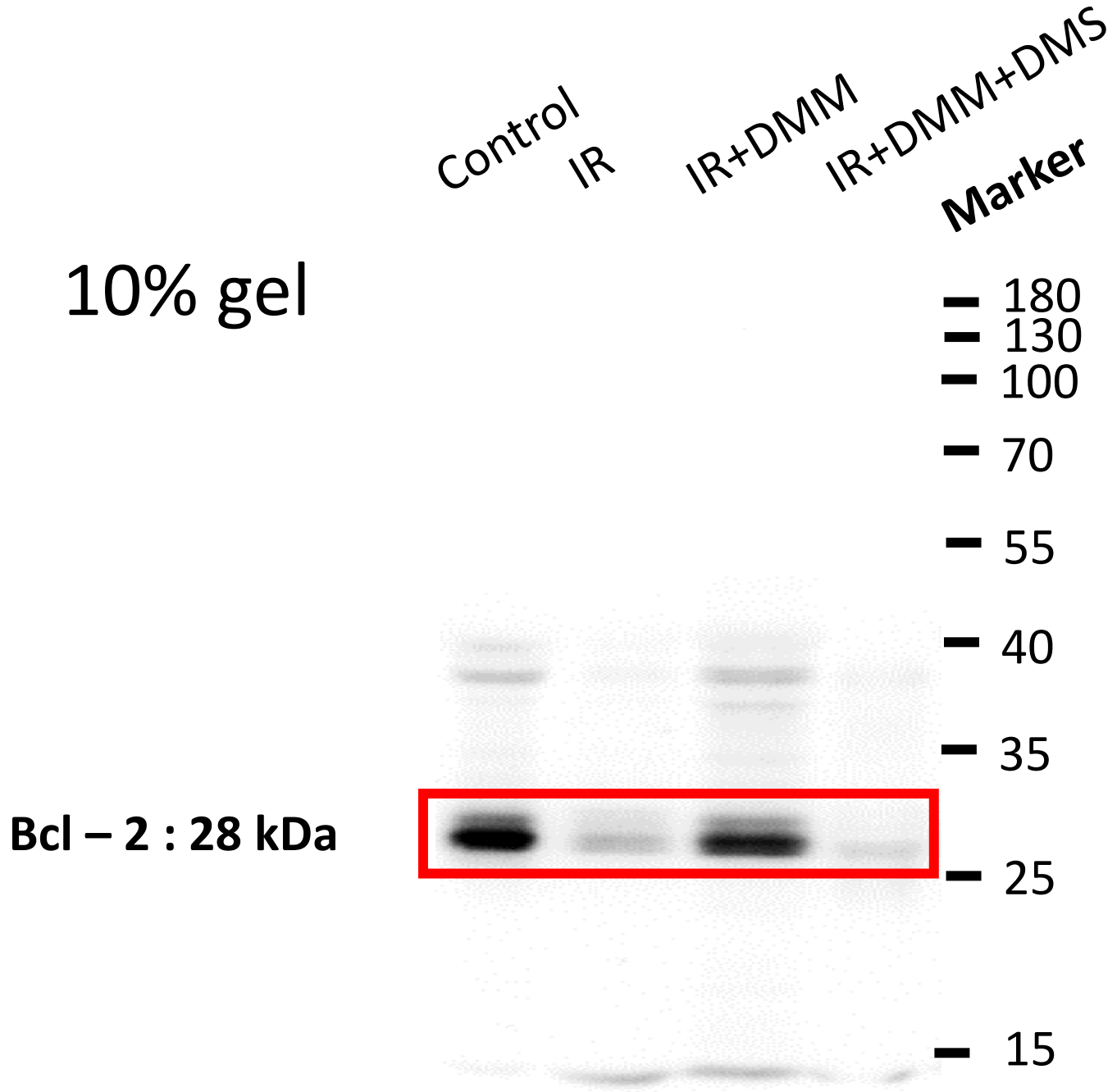

10% gel
Control
IR
IR+DMM
IR+DMM+DMS
Marker
180
130
100
70
55
40
35
25
15
Bcl – 2 : 28 kDa

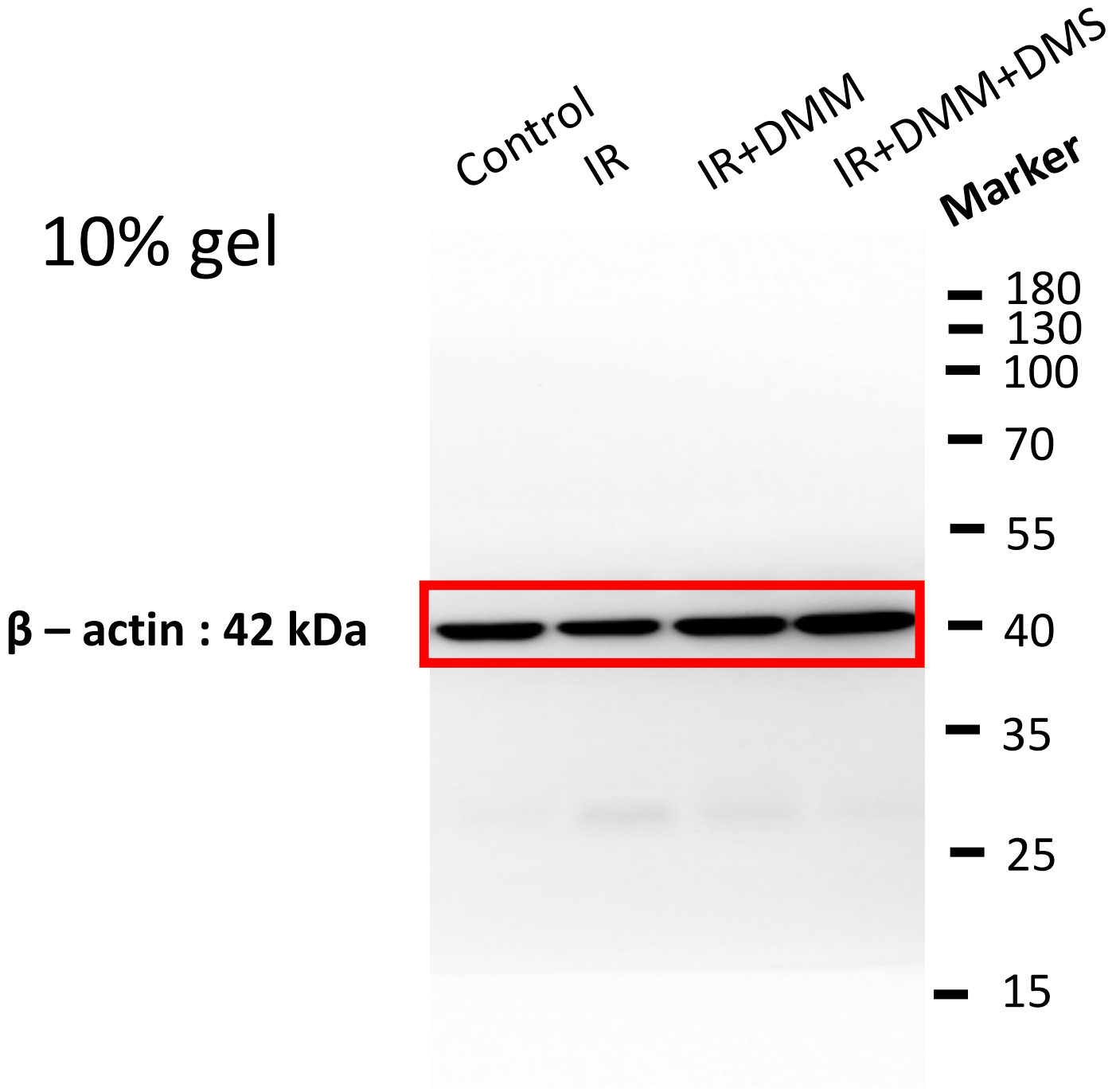

10% gel
Control
IR
IR+DMM
IR+DMM+DMS
Marker
180
130
100
70
55
40
35
25
15
β – actin : 42 kDa

Lung tissue

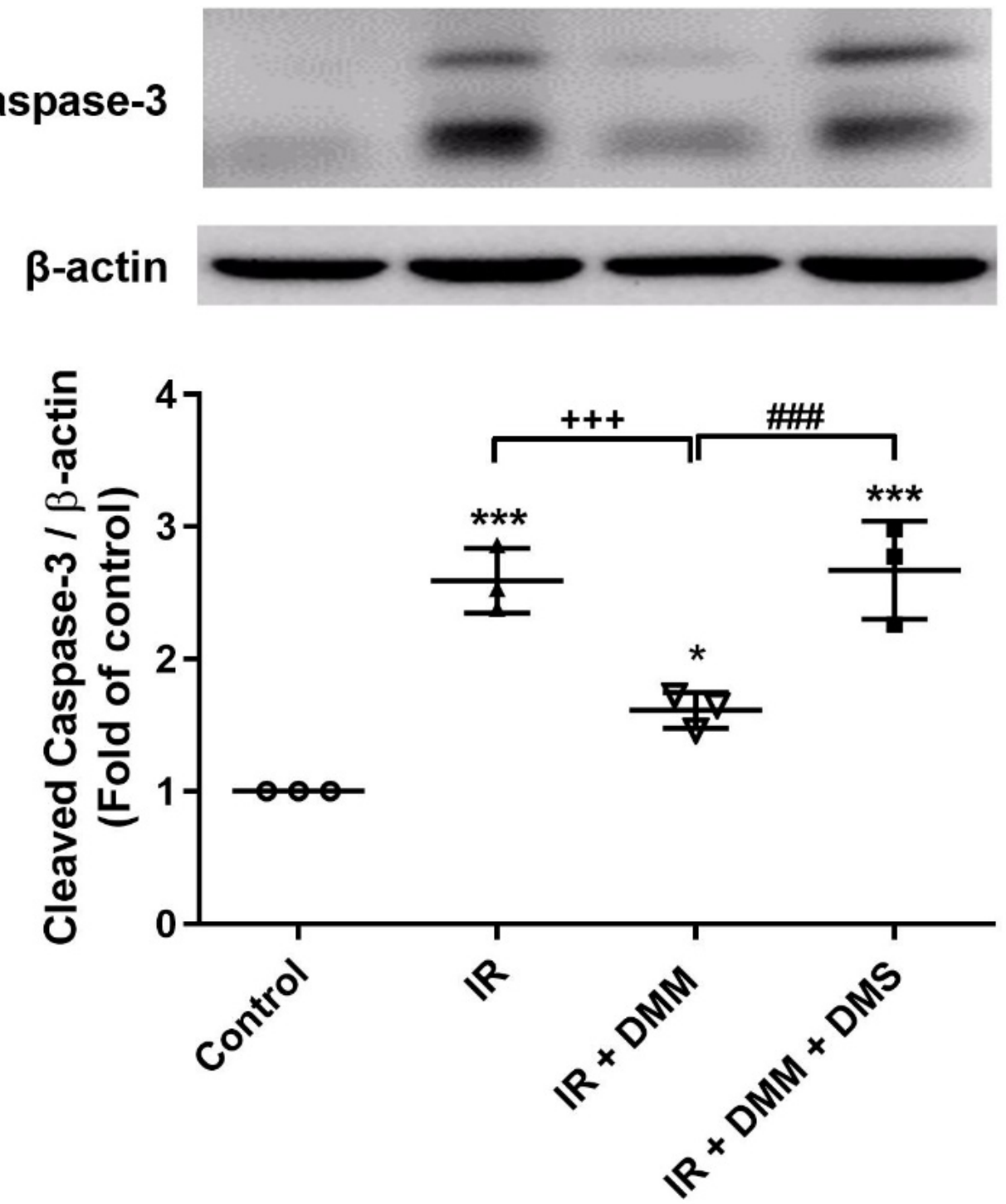
Cleaved caspase-3
β-actin
Cleaved Caspase-3 / β-actin
(Fold of control)
4
3
2
1
0
+++
###
***
*
***
Control
IR
IR + DMM
IR + DMM + DMS

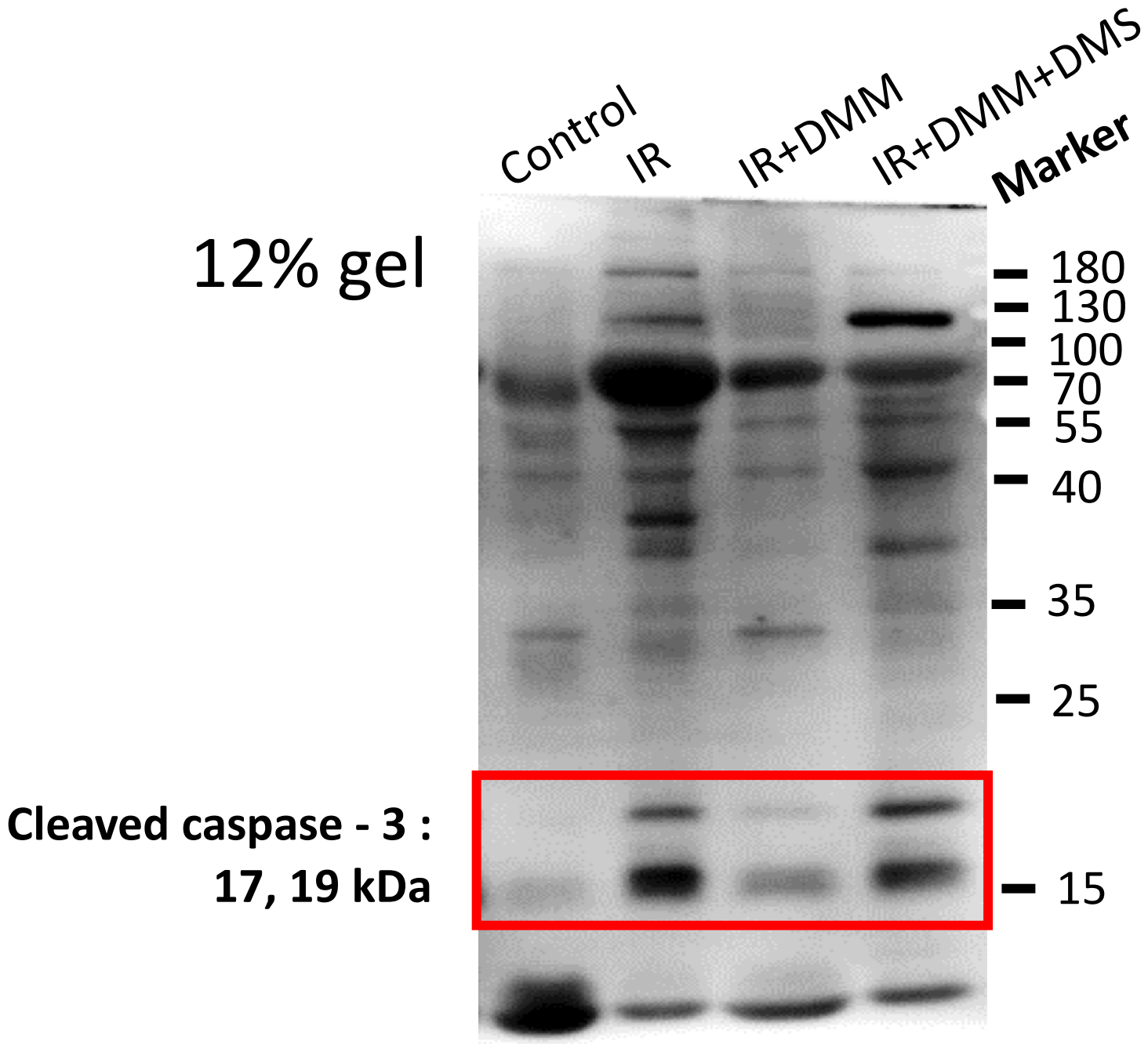
Control
IR
IR+DMM
IR+DMM+DMS
Marker
12% gel
180
130
100
70
55
40
35
25
15
Cleaved caspase - 3 :
17, 19 kDa

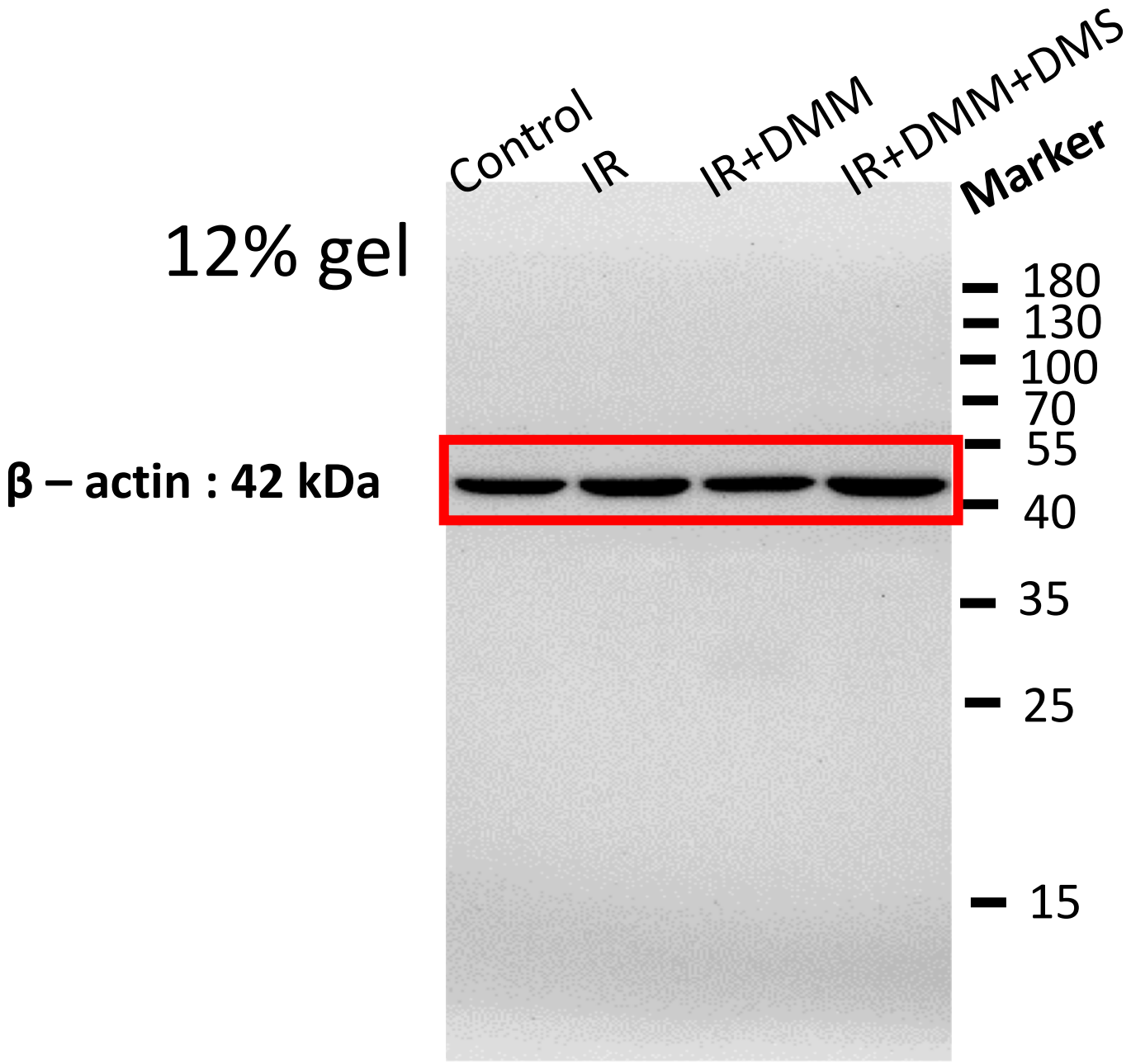
Control
IR
IR+DMM
IR+DMM+DMS
Marker
12% gel
180
130
100
70
55
40
35
25
15
β – actin : 42 kDa

MLE-12 cell line

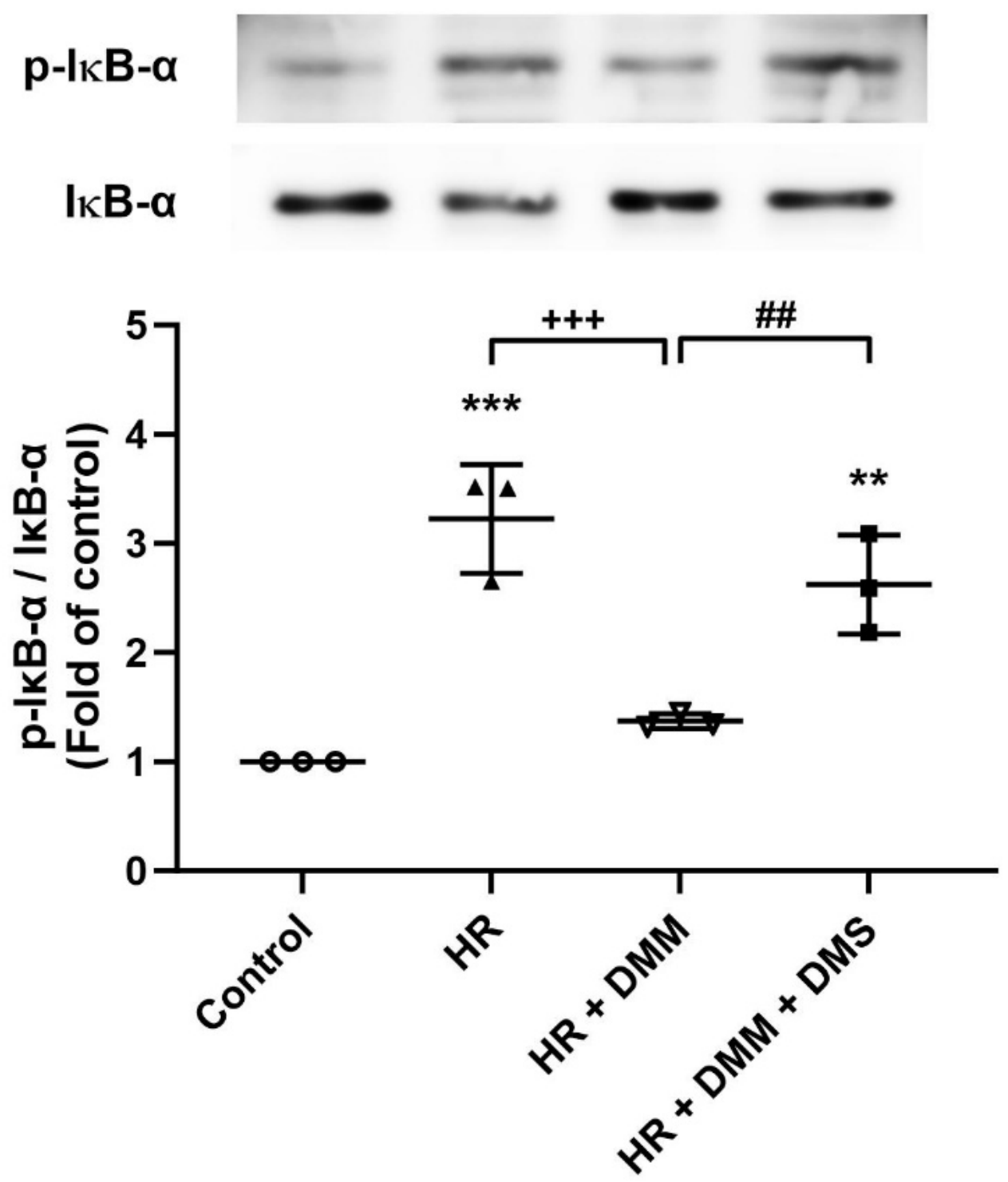

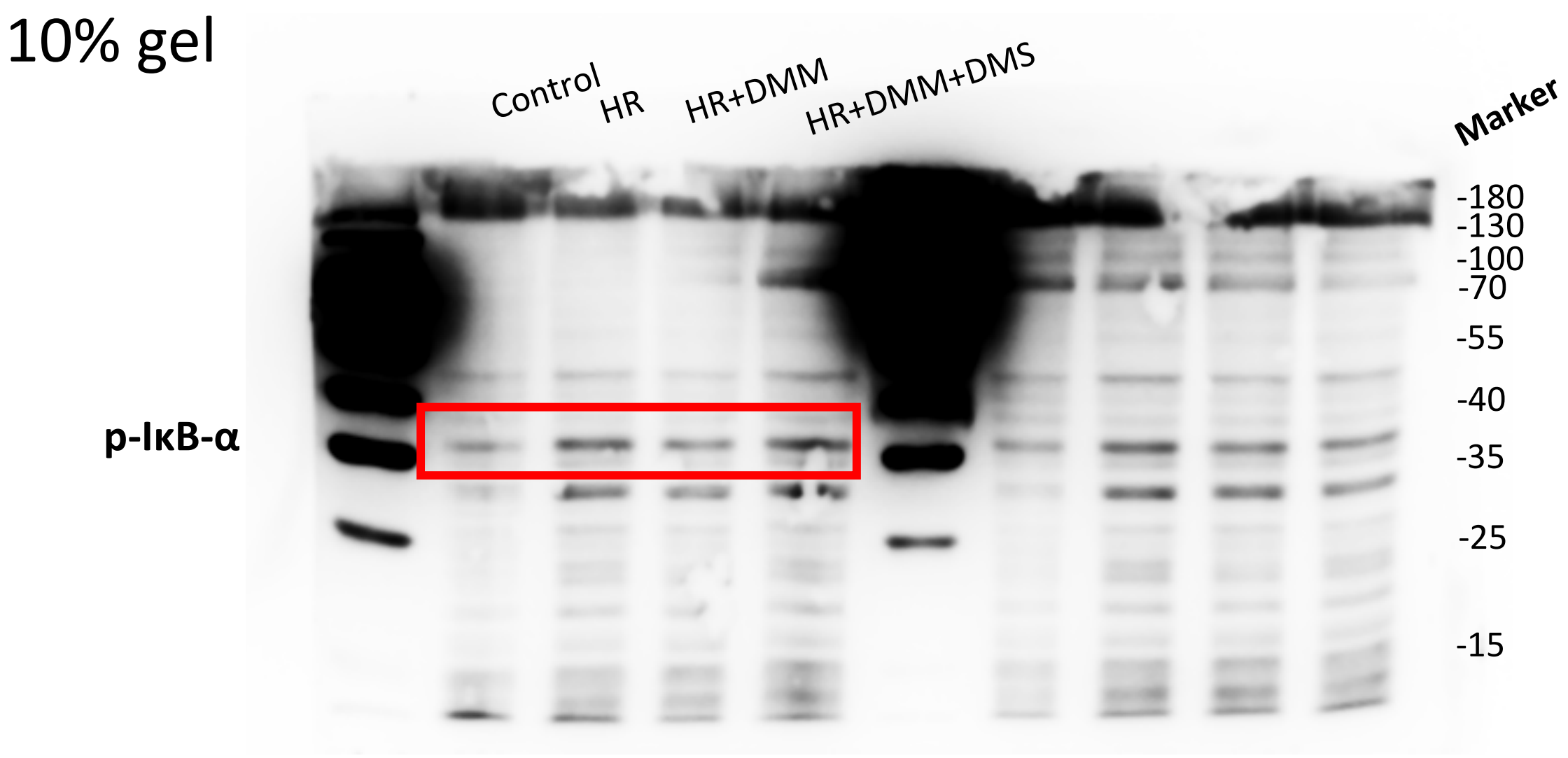

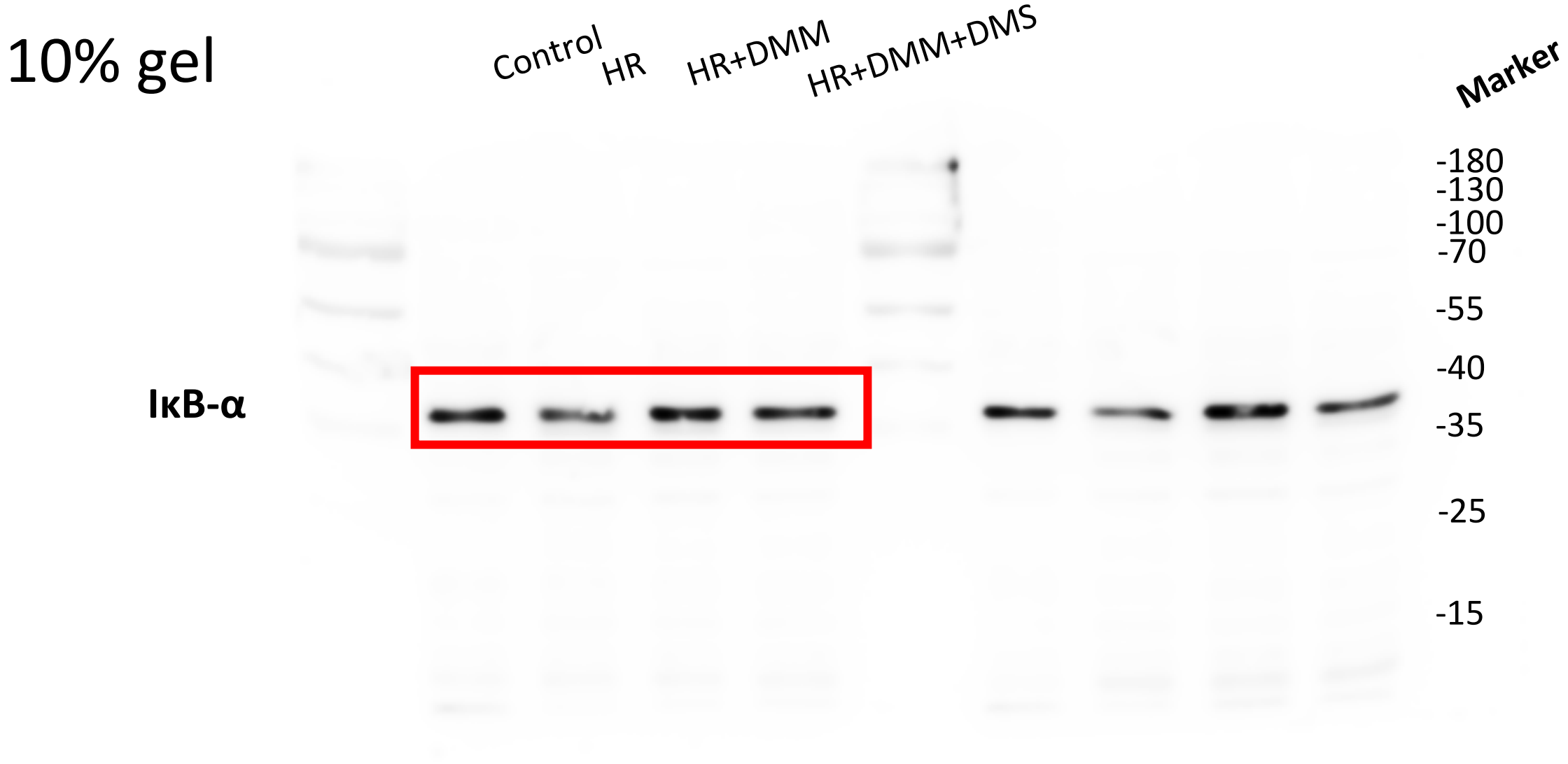

MLE-12 cell line

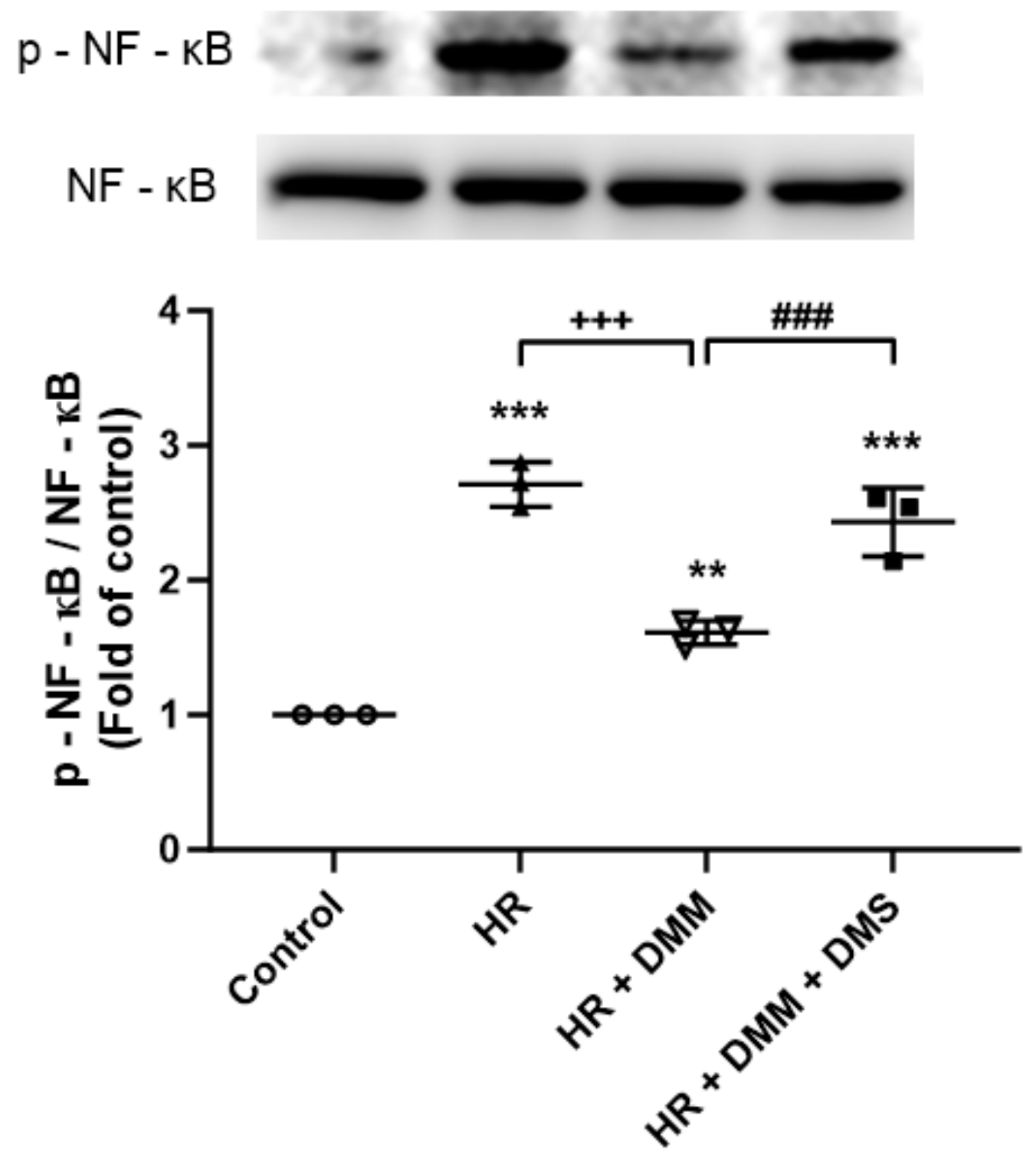

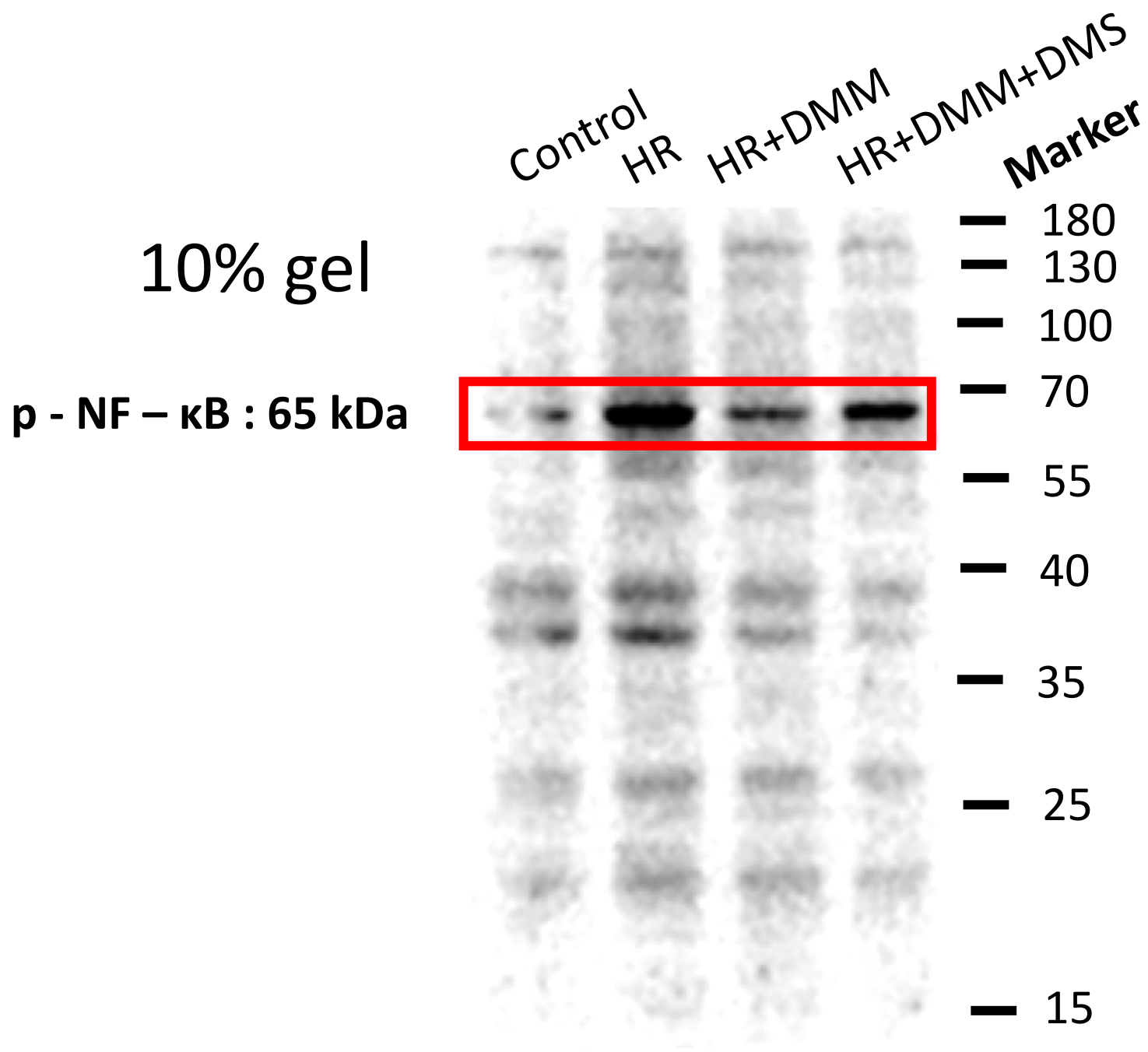

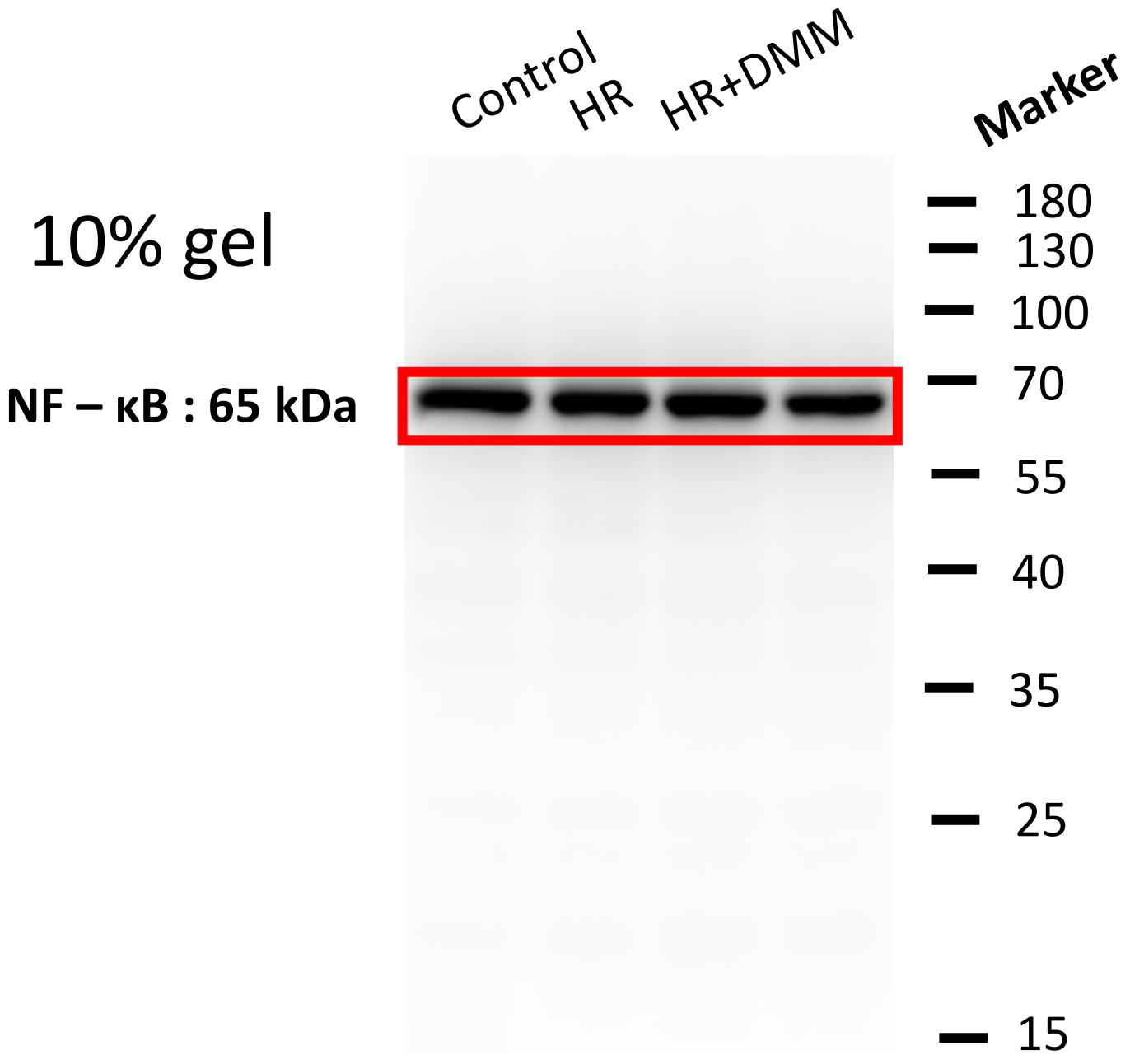

Supplement: Supplementary file 1 [file DataSheet1.pdf]
